# Techno-Economic Assessment for Methane Mitigation through Catalytic Oxidation from Low-Concentration Sources

**DOI:** 10.1021/acs.iecr.6c02071

**Published:** 2026-10-21

**Authors:** Devesh Sathya Sri Sairam Sirigina, Sai Gokul Subraveti, Simon Roussanaly, Rahul Anantharaman, Shareq Mohd Nazir

**Affiliations:** † Department of Chemical Engineering, 7655 KTH Royal Institute of Technology, Stockholm 11428, Sweden; ‡ 196914 SINTEF Energy Research, Trondheim 7034, Norway

## Abstract

Mitigating methane emissions is crucial to achieving climate targets by limiting the temperature rise by the end of the century. This study presents techno-economic estimates for methane mitigation from low-concentration sources by two catalytic routes: thermal and photocatalytic oxidation. Methane mitigation costs for inlet concentrations representative of ambient air (2 ppm), waste stream from an air separation unit (4 ppm), air close to wetlands (40 ppm), ventilation stables (300 ppm—highest reported concentration in the literature for stables), and manure storage (1 vol %) are reported. Mitigation costs (using GWP100:28) are inversely proportional to the inlet methane concentration and range between 52 and 186,000 $/t-CO_2_eq in the thermal route and 48–239,000 $/t-CO_2_eq in the photocatalytic route for the concentration ranges in this study. We studied the effect of different process-level characteristics on methane mitigation costs. Among other process characteristics, cheap moisture-resistant catalysts and cheap electricity can bring down the costs in the thermocatalytic route, while the availability of sunlight and higher catalytic activity are necessary for photocatalytic routes. Emissions related to electricity use will result in higher methane mitigation costs. Since methane is a short-lived gas in the atmosphere and gets converted to CO_2_, the assumption around the time frame for global warming potential will also affect the mitigation costs if the costs are reported per t-CO_2_eq mitigated.

## Graphical abstract

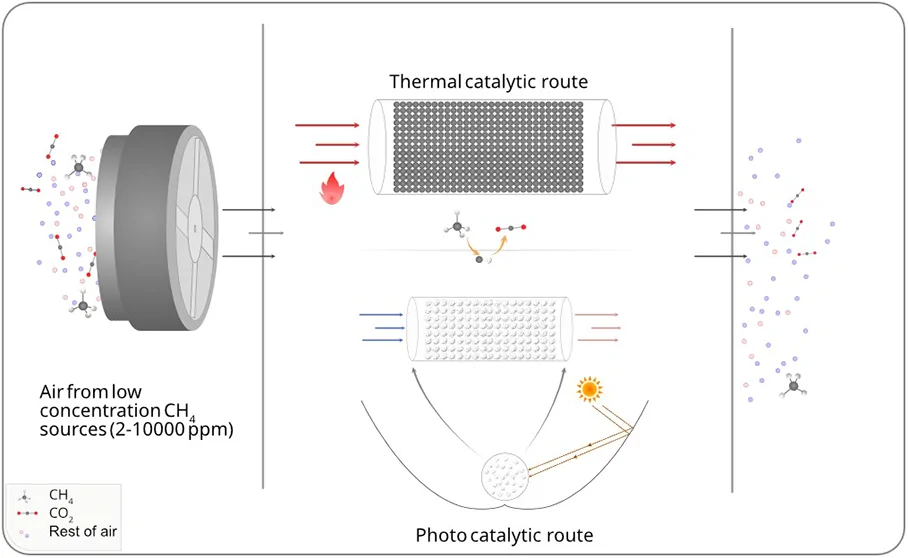

## 1 Introduction

Methane is a potent greenhouse gas (GHG) contributing to about 18% of global net anthropogenic greenhouse gas emissions in carbon dioxide equivalents.^1^ Wetlands and freshwaters account for more than 80% of natural methane emissions, while ruminants contribute more than 30% of total anthropogenic emissions followed by oil and gas industry, landfill and waste, coal mining, and rice cultivation^2^ The Intergovernmental Panel on Climate Change (IPCC) states that the most ambitious warming scenario of limiting global temperature rise to 1.5 °C will require 44% reduction (relative to 2019) in methane emissions by 2040.^3^ Moreover, deep reductions in methane emissions can help rapidly reduce warming because of its short-lived nature, thereby helping to prevent temperature overshoot.^3^ Measures for methane mitigation from the most prominent sources such as agriculture and fossil-based energy-related activities have been presented in the literature.^4–7^ Some of the common measures that are explored for methane mitigation from agriculture include reducing emissions from enteric fermentation. These measures include the use of feed additives, direct inhibitors, and similar measures aimed at improving rumen fermentation efficiency.^7^ However, these measures have limited mitigation potential and involve a change in current agricultural practices. Another approach is treating the methane emissions closer to their source of emissions or from ambient air. Ming et al.^8^ presented a review of methods to remove methane directly from the atmosphere. Nisbet et al.^9^ presented several aspects surrounding methane mitigation, namely, identifying the sources, quantification, and the strategies for emissions that can easily be addressed and also the otherwise hard-to-mitigate emissions. According to the biofilter concepts presented by Nisbet et al.,^9^ air with methane concentrations between 10 and 100 ppm extracted from a barn is sent to the soil cultured with methanotrophs in a greenhouse. However, an issue with biofilters is the formation of nitrous oxide due to the presence of ammonia in the gaseous mixture.^9^ Additionally, the rate of methane conversion was reported to be slow in the biofilters due to its low water solubility.^10,11^ As a result, at lower gaseous concentrations such as found in the ambient air or from several agricultural sources, larger biofilter volumes are required.^10^ This was also shown in the study by Pennacchio et al.,^12^ who examined and presented constraints for several ambient methane removal systems. The study noted that among other systems, large volumes of removal systems for biofilters are needed for ambient methane removal. Despite the well-known photoactivity of TiO_2_ on methane oxidation, studies with photocatalysis of the barn air in a separate reactor have not reported conversion of methane.^9^ A pilot-scale study using UV-A light and a special photocatalyst did not show oxidation of methane.^13^ A similar study on the exhaust air from a swine barn did not observe significant reduction in methane concentrations.^14^ The reason is the low selectivity of OH radicals toward methane in the presence of more reactive volatile organic compounds (VOCs).^9^

Compared to CO_2_ capture, methane mitigation via oxidation from ambient and very low-concentration sources is sparsely researched, especially in terms of process and techno-economic analysis. Hickey and Allen^15^ presented the cost of enhanced methane oxidation from atmospheric air by thermal catalytic, photocatalytic, capture-based oxidation, and biofiltration. The estimated costs for treating methane present in ambient air by thermal catalytic, photocatalytic, and capture-based oxidation, respectively, are 3 M€/tCH_4_ (107 k€/t-CO_2_eq), 82 k€/tCH_4_ (2930 €/t-CO_2_eq), and 3900 €/tCH_4_ (139 €/t-CO_2_eq), using a global warming potential of 28 for CH_4_. Ming et al.^8^ provided perspectives on several methane removal methods from low-concentration and ambient sources and presented the enhancement of the natural chlorine sinks in the atmosphere as one of the cheapest methods.

Sirigina et al.^16^ presented process concepts to mitigate methane and carbon dioxide in a coupled system. Methane mitigation by thermal- and photocatalytic oxidation routes, with and without CO_2_ capture, was presented. Integrated processes combining methane oxidation (and N_2_O decomposition in later studies) and CO_2_ capture can exploit the synergies between the individual processes, for example, heat integration between CH_4_ oxidation and CO_2_ capture. Catalytic processes can be integrated with existing CO_2_ capture to mitigate multiple greenhouse gases. A sorbent-based direct air capture was considered for the CO_2_ capture after methane oxidation. This work discussed process performance and identified key parameters and design conditions to lower the energy penalty per unit carbon dioxide equivalent mitigated from various configurations.^16^ The present work builds upon this and estimates the cost of methane mitigation from low-concentration sources via thermal- and photocatalytic oxidation routes. Further, this study considers the necessary components and design conditions in a continuous process to mitigate methane from low-concentration sources and ambient air. Steady-state process models simulating methane mitigation using thermal and photocatalysts were developed, and the cost of methane mitigation is presented for treating ambient air and emission sources with higher levels of concentration (up to 1 vol %). Sensitivity analysis is carried out to identify key variables and design conditions influencing the mitigation cost, including the effect of assuming different Global Warming Potential (GWP). This work uses “mitigation” as the term for methane management, and the discussion about whether scenarios correspond to methane removal or methane emission avoidance is out of scope of this study.

## 2 Process Concept Description and Methodology

### 2.1 Methane Concentrations Considered in This Study

Methane oxidation as a mitigation option is presented for 5 selected sources of methane emissions and their respective concentration levels as shown in Table 1.

**Table 1:** Methane Sources and Their Representative Concentrations^a^

**Source**	**Concentration (ppm)**	**Reference**
Ambient air	2	-
Waste stream from air separation unit (assuming no methane in the product stream)	4	^17^
Air above wetlands	40	^18^
Ventilation stables in agricultural farms (maximum concentration reported in literature)—considered as the reference case in this study	300	^19^
Concentration above manure storage	10,000	^19^

a Methane concentration in manure storage headspace can be up to 2.8 vol %,^19^ but a representative value of 1% was considered in this study.

### 2.2 Process Description

#### 2.2.1 Thermal Catalytic Oxidation Route

The thermal catalytic route incorporates catalysts to aid methane oxidation, usually at lower temperatures (<400 °C). Most of the catalysts identified and reported in the literature can oxidize methane at temperatures between 300 and 700 °C.^20^ The design conditions considered in this study are based on a 6.5 wt % Pd/Al_2_O_3_ catalyst. The experimental performance of the catalyst was reported by Alyani and Smith.^21^ The catalyst was tested for methane concentrations of 1000 ppm (dry conditions) and 5000 ppm (both dry and wet conditions) and was shown to be active for methane oxidation at 330 °C. However, the catalyst exhibited reversible inhibition by moisture present in the stream, which is typical of PdO-based catalysts.^21–24^ A dehumidification step is necessary to reduce the inhibitory effect of moisture on the catalyst. Modeling assumptions for the process have already been discussed in Sirigina et al.^16^

The process schematic for thermal catalytic methane mitigation is shown in Figure 1. A steady-state model of the process was developed in Aspen Plus V12. The feed air is representative of low-concentration streams with methane concentration equal to or higher than the ambient concentration. In this study, the upper limit for methane concentration of 300 ppm from dairy ventilation stable reported in Stolaroff et al.^19^ is considered as the reference case. This is only a representative methane concentration to assess the process and techno-economic performance of the proposed methane mitigation routes and compare it for different methane concentrations. The volumetric flow rate of the inlet stream is 1400 m^3^/min. The temperature of the inlet stream is assumed to be 15 °C and has a relative humidity (RH) of 60%. First, an inlet blower (Blower in Figure 1) is required to overcome the pressure drops in different components of the process. Pressure drop estimation and related assumptions are presented in the Supporting Information. The stream from the blower is dehumidified before being sent to the reactor. A heat exchanger (Recuperator in Figure 1) recovers the heat from the outlet stream of the reactor. The feed stream entering the reactor (Thermal catalytic reactor in Figure 1) is heated to 330 °C to drive the thermal catalytic oxidation.^21^ The design temperature considered in the reactor is specific to the catalyst considered in this study, 6.5 wt % Pd/Al_2_O_3_, and it was chosen based on the experimental results available in the literature.^21^ The sensible heat remaining in the hot outlet stream from the recuperator can be used, for example, in district heating network.

**Figure 1:**
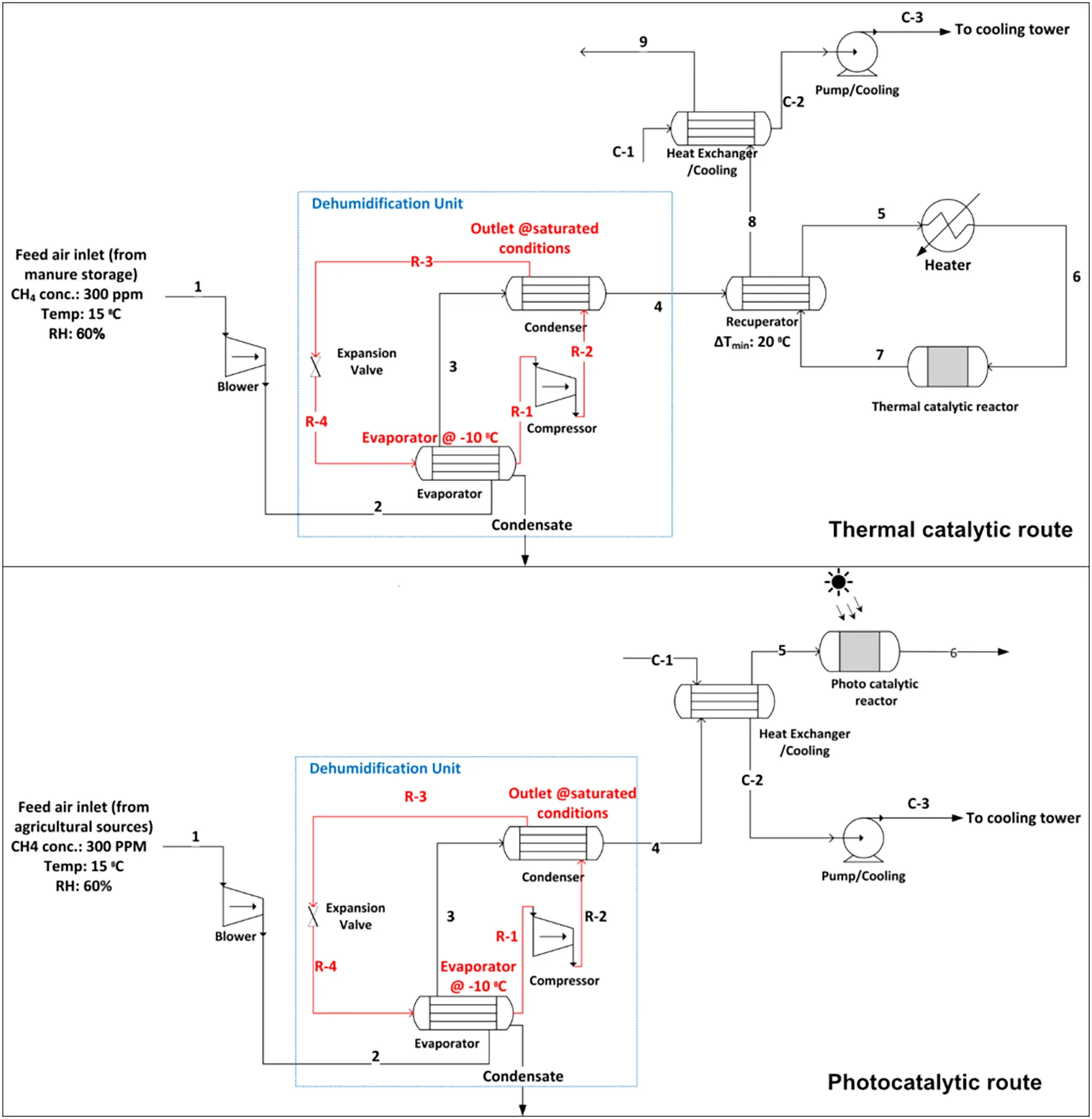
Process schematic of methane mitigation by thermal and photocatalytic routes.

#### 2.2.2 Photocatalytic Oxidation Route

The process schematic of methane mitigation by photocatalytic oxidation is also shown in Figure 1. The inlet conditions of the stream were set at 15 °C, 1 atm, and with a volumetric flow rate of 1500 m^3^/min. The composition of inlet stream is the same as considered in the thermal catalytic route. Photocatalysts use solar energy to drive the oxidation reaction. The catalyst 0.8 wt % CuO/ZnO was chosen as the catalyst in this study due to its superior photoactivity.^25^ The same process configuration as the thermal catalytic route was adopted here: a blower moves the air into the system, which then goes through a dehumidification step and a cooler, followed by a photocatalytic reactor for methane oxidation.^16^ The cooling step ensures the stream is at ambient temperature for the photocatalytic reaction. The effect of the hot stream entering the photocatalytic reactor has not been considered in this study. The process model was simulated using Aspen Plus v12 with the assumption that the reactor is supplied with the solar irradiation necessary to carry out the reaction. RStoic model with the fixed conversion (92.5%) was used to simulate the photocatalytic reactor. The conversion of 92.5% used in this study was according to the maximum conversion observed in experiments with the 0.8 wt % CuO/ZnO catalyst as reported in literature.^25^ The design of the dehumidifier is based on the same methodology as used in the thermal catalytic route. The material and energy flows obtained from process modeling were used in techno-economic assessment (TEA) to estimate the specific cost of mitigation. The methodology for TEA is described in the following section.

### 2.3 Methodology and Assumptions for Techno-Economic Assessment

The overall methodology for economic assessment is based on the guidelines presented in Roussanaly et al.^26^ The specific mitigation cost is estimated using eq 1, which is a function of Total Capital Requirement (TCR), interest/discount rate (*r*), lifetime of the plant (*n*), Variable Operating and Maintenance (VOM) costs, Fixed Operating and Maintenance (FOM) costs and the revenue. The global warming potential (GWP) for methane is considered as 28 in this study (for a 100-year time frame—GWP100).^27^ GWP100 was chosen primarily because of its widespread use. This enables easier comparison of the results with that of the results present in the literature for similar technologies. However, the authors acknowledge the shortcomings of using simplified metrics such as GWP100 or GWP20, which either ignore short-term warming impacts such as the near-term temperature overshoot leading to irreversible tipping points,^28^ or long-term effects such as the end-of-century temperature targets. Some of the results were also discussed by presenting them with GWP20, thereby highlighting that the engineering costs of cutting methane emissions will look more effective if we consider their high near-term warming impact. The GWP value was based on Assessment Report 5 (AR5) for nonfossil methane emissions. This is consistent with most of the methane emission sources considered in this study, which are biogenic in origin (Wetlands, Ventilation stables in agricultural farms, Manure storage).

1 $$\mathrm{specific cost}\left(\frac{\$}{t-CO_{2}\mathrm{eq.}}\right)=\frac{(TCR\cdot \left(\frac{r{\left(1+r\right)}^{n}}{{\left(1+r\right)}^{n}-1}\right)+\left(VOM+FOM\right))\left(\frac{\$}{\mathrm{year}}\right)-\mathrm{Revenue}\left(\frac{\$}{\mathrm{year}}\right)}{CO_{2}\;\mathrm{equivalent mitigated from the process}\left(\frac{t-CO_{2}\mathrm{eq.}}{\mathrm{year}}\right)}$$

Table 2 ^29^ shows the methodology adopted to estimate the TCR. All the costs have been adjusted to 2023 using Chemical Engineering Plant Cost Index (CEPCI).^30^

**Table 2:** Methodology in Estimating Total Capital Requirement

**Capital Cost component**	**Estimation**
Bare erected cost (BEC)	Sum of installed cost of all the equipment
Engineering procurement construction costs (EPCC)	0.1* Bare erected cost
Process contingency	0.4* Bare erected cost
Project contingency	0.225*(BEC + EPCC+ Process contingency)
Total contingency	Process contingency + Project contingency
Total plant costs (TPC)	BEC + EPCC + Total contingency
Owner’s costs	0.202*TPC
Total overnight costs (TOC)	TPC + Owner’s costs
Total capital requirement (TCR)	1.14*TOC

Operating and maintenance (O&M) costs are subdivided into variable and fixed components. The costs of electricity, cooling water makeup, and catalyst are included in the variable O&M costs. The fixed O&M costs include costs for operating labor, maintenance, support and administrative labor, insurance, and property taxes. The unit cost details and the calculation methodology are shown in Table 3.

**Table 3:** Methodology and Unit Costs for Operating and Maintenance Expenses

**Component**	**Cost**	**Unit**	**Reference**
Electricity cost	65	$/MWh	^31^
Cooling water makeup costs	0.39	$/m^3^	^29^
Operating labor	18	$/h	-
Maintenance, support, and administrative labor	2.5% of TOC	-	^29^
Insurance and property taxes	2% of TOC	-	^29^

#### 2.3.1 Assumptions in Thermal Catalytic Route

The material and energy flows for the techno-economic assessment were obtained from process models (simulated using Aspen Plus) for the respective cases. The equipment cost data for most of the process equipment were taken directly from the Aspen Process Economic Analyzer (APEA). For some of the equipment, the cost was estimated using cost equations from the literature. The data source and the relations used are shown in Table 4. Total installed cost was then estimated using installation cost factors obtained from Peters et al.^32^ Installation costs include foundations, supports, labor, and other factors related to the installation of the purchased equipment.^32^

**Table 4:** Methodology to Estimate the Purchase Cost of Process Equipment

**Equipment**	**Equipment purchase cost**	**Installation factor/Relation**	**Reference**
Blower	Equipment costs by scaling. Scaling exponent: 0.59	1.45	Reference blower cost for scaling from ref 33; Exponent and installation factor from ref 32
Compressor (dehumidifier)	Purchased equipment cost = log_10_(W) + 0.03867 * W^2^ + 446.7 * W + 1.378 * 10^5^; W = Compressor work in kW	1.6	Reference for compressor from ref 34; installation factor from ref 32
Heat exchangers	APEA	1.45	^32^
Pumps	APEA	APEA	-
Electric heater	Specific cost: 140 $/kW_elec_	1.45	Specific cost from ref 35; installation factor from ref 32
Reactor	Cost estimation for a tubular heat exchanger	1.6	^32^

The sizing of the reactor is based on the amount of catalyst required for the conversion. The mass of catalyst is estimated based on the steady-state mole balance relation given in Alyani and Smith.^21^ The mole balance equation is given by

2 $$\frac{dX_{CH_{4}}}{d\left(\frac{W}{F_{CH_{4}}^{o}}\right)}=\frac{{\eta k}_{s}{C_{T}}^{2}P\left(1-X_{CH_{4}}\right)y_{CH_{4}}^{o}}{1+K_{H_{2}O}P\left(\alpha_{H_{2}O}+2X_{CH_{4}}\right)y_{CH_{4}}^{o}}$$

*X*_CH_4__ in eq 2 represents the conversion, which is assumed to be 99% in this work. The catalyst amount necessary for the conversion at 330 °C is scaled according to eq 2 using the kinetic data reported in Alyani and Smith.^21^ This maximum conversion is intended to represent the best-case scenario for the costs with the assumed catalyst in this study. *W* is the mass of catalyst, *F*_CH_4__^o^ is the molar feed flow rate of methane, P is the total pressure, α_H_2_O_ is the partial pressure ratio of H_2_O to CH_4_ at the inlet of the reactor, and *C*_T_ represents the total number of active sites on the catalyst. *k*_s_ and *K*_H_2_O_ represent the rate constant for methane conversion and the equilibrium constant for H_2_O adsorption, respectively, and are given by eqs 3 and 4 ^21^

3 $$k_{s}={k_{s}}^{o}\exp\left[\frac{-E_{a}}{R}\left(\frac{1}{T}-\frac{1}{T_{O}}\right)\right]$$

4 $$k_{H_{2}O}={k_{H_{2}O}}^{o}\exp\left[\frac{-\Delta H_{H_{2}O}}{R}\left(\frac{1}{T}-\frac{1}{T_{O}}\right)\right]$$

The estimated values of the constants *k*_s_ and *k*_H_2_O_ at 330 °C are 13.5 $\frac{mol\cdot g_{cat}}{{mol_{site}}^{2}\cdot Pa.s}$ and 5.32 × 10^–3^ Pa^–1^, respectively.^21^ Using the porous catalyst density (1.488 g/cm^3^ ^21^) and assuming a bed porosity of 0.4, the required reactor volume is estimated. For an isothermal reactor, the cost can be approximated using a shell-and-tube heat exchanger. The volume inside the exchanger tubes is the reactor volume. With a 2-in. outer diameter, the tube thickness was estimated using design equations for cylindrical shells based on the American Society of Mechanical Engineers (ASME) boiler and pressure vessel code.^32^ The maximum allowable working stress for stainless steel 304 is 72,400 kPa, and considering a joint efficiency of 0.6, the thickness is estimated at approximately 3 mm. With a tube length of 20 ft, the total heat transfer area is estimated. Using the heat transfer area, the purchased cost of a floating-head heat exchanger for the lowest pressure, for carbon steel, and the given outer diameter is estimated from the graphical data given in Peters et al.^32^ Appropriate adjustment factors for tube diameter and length, exchanger material, pressure, and time index were considered to calculate the final purchase cost. An overall heat transfer coefficient value is used to estimate the size of heat exchangers. More information on the overall heat transfer coefficient is found in the Supporting Information.

The cost of catalyst (Pd/Al_2_O_3_) is assumed to be 811 $/100g.^36^ The lifetime of the catalyst is assumed to be 20 years. The cost of catalysts is converted to an annuity value over the lifetime of the plant.

Electrical energy demand was estimated directly from simulations. Any thermal energy demand in the process is assumed to be fulfilled by equivalent electricity. The temperature of the outlet hot stream from the recuperator is close to 85 °C. The remaining sensible heat in this stream is assumed to be valorized for the 80/40 °C district heating system.^37^ An approach temperature of 5 °C is considered to supply heating to the district heating network. The cost of waste heat supply is 18.4 $/MWh. The economic lifetime of the plant is 20 years, and the weighted average cost of capital (WACC) is 10%.

#### 2.3.2 Assumptions in Photocatalytic Route

The TEA for the photocatalytic methane oxidation route follows a similar methodology as described for the thermal catalytic route. The capital expenditure required for most of the equipment was obtained directly from APEA integrated within Aspen Plus. Material and energy flows estimated from the process models were used to estimate operating expenses. The economic lifetime of the plant was assumed to be 20 years. A capacity factor of 0.48 was considered in the analysis. The capacity factor reflects the hours of solar irradiation (the availability of either diffuse or direct normal irradiation or both, i.e., global horizontal irradiation) available in a meteorological year. The analysis considers Stockholm to be the location. It is thereby assumed that the presence of global horizontal irradiation is enough to drive the photocatalytic oxidation of methane. The considered capacity factor presents an optimistic upper limit for the locations. The lifetime of the catalyst is 20 years, in line with the economic lifetime of the plant. The price of CuO/ZnO catalyst is 10 $/kg.^38^

The photocatalytic reactor, modeled as a compound parabolic collector (CPC), is shown in Figure 2. CPCs are solar collectors with curved reflectors on both sides to concentrate the incoming radiation on a receiver. CPCs can be designed to capture and concentrate both the direct and diffuse components of inlet UV in solar radiation onto the receiver. The reflected radiation is distributed across much of the perimeter of the receiver filled with photocatalysts.^39^ CPCs were studied and were found to be suitable for applications involving water treatment using solar energy and photochemical applications.^39–41^ Considering the similarities of the latter in structure and operation to the photocatalytic reactor in this work, a CPC-type solar collector is considered as a photocatalytic reactor. However, in this study, emphasis was not placed on the criteria for choosing solar collector technology over another technology. Therefore, the choice of CPC as a photocatalytic reactor is preliminary, and it is intended to highlight the costs of photocatalytic methane oxidation in comparison to the thermal catalytic route presented in this study. The CuO/ZnO nanocomposite photocatalysts are filled inside the fixed-bed photoreactor. The bulk density of the nanocomposite photocatalysts was assumed to be 833 kg/m^3^.^25^ A CPC with a receiver of 26 mm in diameter, 2.5 mm in thickness, and 380 mm in length was considered. An acceptance angle (rays with higher incident angle than this are not collected by the receiver) of 45° with a concentration factor (ratio of flux collected by the receiver to the actual flux available from the sun) of 1.41, aperture width of 11.55 cm, and a total collector surface area of 1000 cm^2^ was considered for the receiver.^42^ The total volume of a reactor tube is 132 cm^3^. The number of collector tubes is estimated based on total reactor volume. The total reactor volume was calculated based on the bulk density of catalyst and the catalyst mass required for the conversion. The catalyst mass was estimated using the following design eq (eq 5) for a packed bed with plug-flow^32^

5 $$\frac{W_{c}}{F_{o}}=\int_{X_{i}}^{X_{f}}\frac{dX}{-r^{\prime }}$$

where *W*_c_ is the mass of catalyst, and *F*_o_, *X*, and −*r*′ are the molar feed rate of the reactant, conversion, and the rate of reaction, respectively. The rate of methane oxidation given by eq 6

6 $$-r^{\prime }=k^{\prime }C_{CH_{4}}$$

To estimate the rate of reaction for a heterogeneous catalytic reaction, the specific rate, *k*′, needs to be known. The first-order specific rate, *k*, of the photocatalyst 0.8 wt % CuO/ZnO for the flow reactor setup used by Li et al.^25^ was estimated to be 0.112 min^–1^.

**Figure 2:**
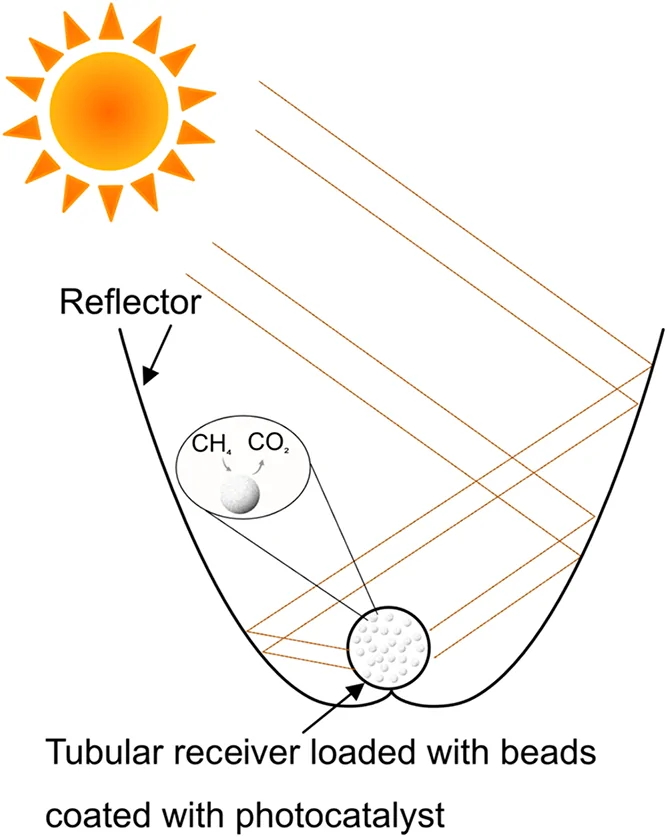
Illustration of CPC with tubular absorber loaded with photocatalysts.

The value of the specific rate *k*, which is available from Li et al.,^25^ was modified to be accommodated in eq 6, which results in eq 7.

7 $$k_{\mathrm{mod}ified}\left(\min^{-1}\right)=k_{original}\frac{\rho_{\mathrm{mod}ified}}{\rho_{original}}$$

The specific rate reported by Li et al.^25^ is due to the large reactor used with the catalyst dispersed at the bottom, while also containing the intrinsic catalytic activity. It is primarily because the rate constant is estimated based on fixed-bed reactor, and since the rate constant is given in time^–1^ (first-order reaction), the constant inherently includes the bulk density $\left(k^{\prime }\left[\frac{m^{3}}{\left(kg_{cat}\cdot s\right)}\right]\cdot \rho \left[\frac{kg}{m^{3}}\right]=k\left[\frac{1}{s}\right]\right)$ . The use of the original rate constant (*k*_original_: 0.112 min^–1^) to estimate the reactor volume would require us to use the original bulk density $\left(\rho_{original}:0.11\left[\frac{kg}{m^{3}}\right]\right)$ for conversion into the mass of catalyst. Alternatively, we can modify the original rate constant based on the bulk density under consideration $\left(\rho_{\mathrm{mod}ified}:833\frac{kg}{m^{3}}\right)$ using eq 7. The modification allows us to account for its change to apply it to the packed bed flow reactors. The result with *k*_modified_ is validated using the reactor data available for a flow system used by Li et al.^25^ to estimate the reactor volume necessary to achieve 92.5% conversion. At 92.5% conversion, the volume of reactor for the given inlet volumetric flow of 15 mL/min was estimated to be 458 mm^3^. The resulting specific rate, *k*_modified_(min^–1^), was used in the above design equation to estimate the volume of the reactor assuming isothermal conditions, neglecting pressure drop, and loss in catalytic activity. The initial rate of reaction under the conditions assumed in this study is 7.3 × 10^–10^ mol/(m^2^ s). The surface oxidation rate estimation follows the assumption that the external mass transfer and internal pore diffusion is fast enough under the modeled conditions. The catalyst mass and the reactor volume were estimated to be 38.6 t and 46 m^3^, respectively. The number of collector tubes required for the total reactor volume is 351858. The cost of reflector and tube are 22.55 €/m^2^ and 10.05 €/m, respectively.^42^ The total cost of parallel CPC reactor setup is calculated using eq 8.^42^

8 cost=tubecost+reflectorcost×collectorarea

## 3 Results and Discussion

Results of techno-economic assessment for methane mitigation by thermal and photocatalytic routes are presented and discussed in the following subsections. Furthermore, a cost-sensitivity analysis with key cost contributors is presented.

### 3.1 Techno-Economic Assessment for the Reference Case

The techno-economic assessment of methane mitigation for the reference case (300 ppm of methane concentration) via two catalytic routes is presented in this section. The specific energy demand for methane mitigation via its oxidation to CO_2_ is 15.2 GJ/t-CO_2_ eq and 8.4 GJ/t-CO_2_ eq in the thermal and photocatalytic routes, respectively. The results in the thermal catalytic route show that the blower requires 40% of the total energy demand, while the dehumidification unit and heater are each responsible for ∼30% of the total energy demand. In the case of the photocatalytic route, the shares of the total energy demand for the blower and the dehumidifier were 53% and 47%, respectively. Material and energy flows feed into the techno-economic assessment to estimate the cost per tonne of CO_2_ equivalent mitigated. The breakdown of costs for the reference case in thermal and photocatalytic routes is shown in Table 5.

**Table 5:** Breakdown of Costs in Thermal and Photocatalytic Routes for Base Case Scenario with 300 ppm of Inlet Methane Concentration

**Cost Component**	**Mitigation costs in thermal catalytic route in $/t-CO**_**2**_ **eq [% of total cost]**	**Mitigation costs in photocatalytic route in $/t-CO**_**2**_ **eq [% of total cost]**
Recuperator	116 [8.9]	- [-]
Cooling pump	2 [0.1]	5 [0.3]
Heater	5 [0.4]	- [-]
Evaporator (dehumidifier)	15 [1.1]	31 [1.9]
Condenser (dehumidifier)	39 [2.9]	53 [3.3]
Reactor	7 [0.5]	247 [15.5]
Compressor (dehumidifier)	26 [2.0]	50 [3.1]
Inlet blower	6 [0.4]	10 [0.6]
Heat exchanger-Cooling	13 [1.0]	14 [0.9]
EPCC	23 [1.7]	41 [2.6]
Total contingency	167 [12.8]	302 [18.9]
Owner’s costs	84 [6.4]	152 [9.5]
**TCR**	**571 [43.7]**	**1031 [64.5]**
Electricity	277 [21.2]	153 [9.6]
Cooling water makeup	0 [0.04]	1 [0.1]
Catalyst	224 [17.2]	23 [1.4]
Operating labor	43 [3.3]	43 [2.7]
Maintenance, support, and administrative labor	107 [8.2]	192 [12.0]
Insurance and property taxes	85 [6.5]	154 [9.6]
**Total cost ($/t-CO**_**2**_ **eq )**	**1308**	**1598**
Revenue from selling waste heat	50	-
**Net cost**	**1258**	**1598**
% CO_2_ equivalents mitigated (ratio of CO_2_ eq avoided from the process with regard to CO_2_ equivalents in the inlet stream to the process)	79	73

The techno-economic assessment for the reference case (300 ppm) in the thermal catalytic route resulted in specific costs of ∼1260 $ per tonne of CO_2_-equivalent mitigated by CH_4_ conversion to CO_2_. This cost estimate also includes a revenue of 50 $/t-CO_2_eq from the waste heat valorization in the district heating network. Total capital expenditure and the operations and maintenance expenditures have a share of 44% and 56% of total cost (excluding revenue), respectively. The recuperator accounts for the largest share of the capital costs due to the large heat exchanger required for the gas–gas heat exchange. Electricity and catalyst-related costs form a major share of operations and maintenance costs.

The specific cost in the reference case (300 ppm) for the photocatalytic route was estimated to be ∼1600 $/t-CO_2_eq. The installed cost of the photocatalytic reactor accounts for ∼15% of the total methane mitigation costs in the photocatalytic route. Among the operations and maintenance costs, since the maintenance, support, and administrative labor costs, and insurance and property taxes are related to capital expenditures, their share in the total methane mitigation costs is high (12% and 10%, respectively). The electricity costs account for 10% of the total mitigation costs, while catalyst-related costs are ∼1% of the mitigation costs. This is very different from the thermal catalytic route, where electricity and catalyst-related costs account for 21% and 17% of the total mitigation costs, respectively. The catalyst assumed in the photocatalytic route (0.8 wt % CuO/ZnO) is cheaper than the catalysts assumed in the thermal catalytic route (6.5 wt % Pd/Al_2_O_3_). The electricity cost difference can be explained by the different capacity factors assumed for the thermal and photocatalytic routes.

The photocatalytic route has a capacity factor of 48% as opposed to 100% for the thermal catalytic route. Designing the photocatalytic process using artificial illumination during low/no sun hours will incur additional costs to the plant due to the added installation and electricity costs; however, it increases the capacity factor. Artificial illumination was not considered in this analysis. The use of artificial illumination may be profitable if the reduced costs due to a higher capacity factor offset the additional installation and electricity costs. CO_2_ equivalents mitigated in the photocatalytic route is almost half that of the thermal route, primarily due to its capacity factor. This increases the overall cost for the photocatalytic route since the capital invested for the equipment is utilized only 48% of the time of the project lifetime. Methane mitigation by the photocatalytic route is cost-intensive relative to the thermal catalytic route in the reference case, despite the number of components in the process being lower and having lower electricity-related costs.

### 3.2 Effect of Inlet Methane Concentration in Different Scenarios on Mitigation Costs

Inlet methane concentration affects both the total energy demand and the amount of catalyst in the reactor, which in turn impacts the total cost per ton of CO_2_ equivalent mitigated. The effect of inlet methane concentration was examined for the selected scenarios presented in Section 2.1. All of the other process parameters were assumed constant from the reference case.

The specific cost of CO_2_ equivalents mitigated through methane oxidation for different inlet methane concentrations in thermal and photocatalytic routes is displayed in Figure 3. In the thermal catalytic route, the cost of mitigation changes from ∼186,000 $/t-CO_2_eq to 52 $/t-CO_2_eq as the inlet methane concentration is changed from 2 to 10,000 ppm. This is mainly due to the large amount of airflow through the system for a specific amount of methane (or t-CO_2eq_) mitigated in the system. A similar trend is observed for the photocatalytic route, where the cost of mitigation changes from ∼240,000 to 48 $/t-CO_2_eq when the methane concentration changes from 2 to 10,000 ppm. Mitigation costs are lower for the photocatalytic route for the 10,000 ppm case when compared to the thermal catalytic route, because the share of the catalyst-related costs is higher in the thermal catalytic route for this case. A cheaper catalyst, like transition metal oxides (e.g., Co-, Fe-based metal oxides), can bring down the costs in the thermal catalytic route.

**Figure 3:**
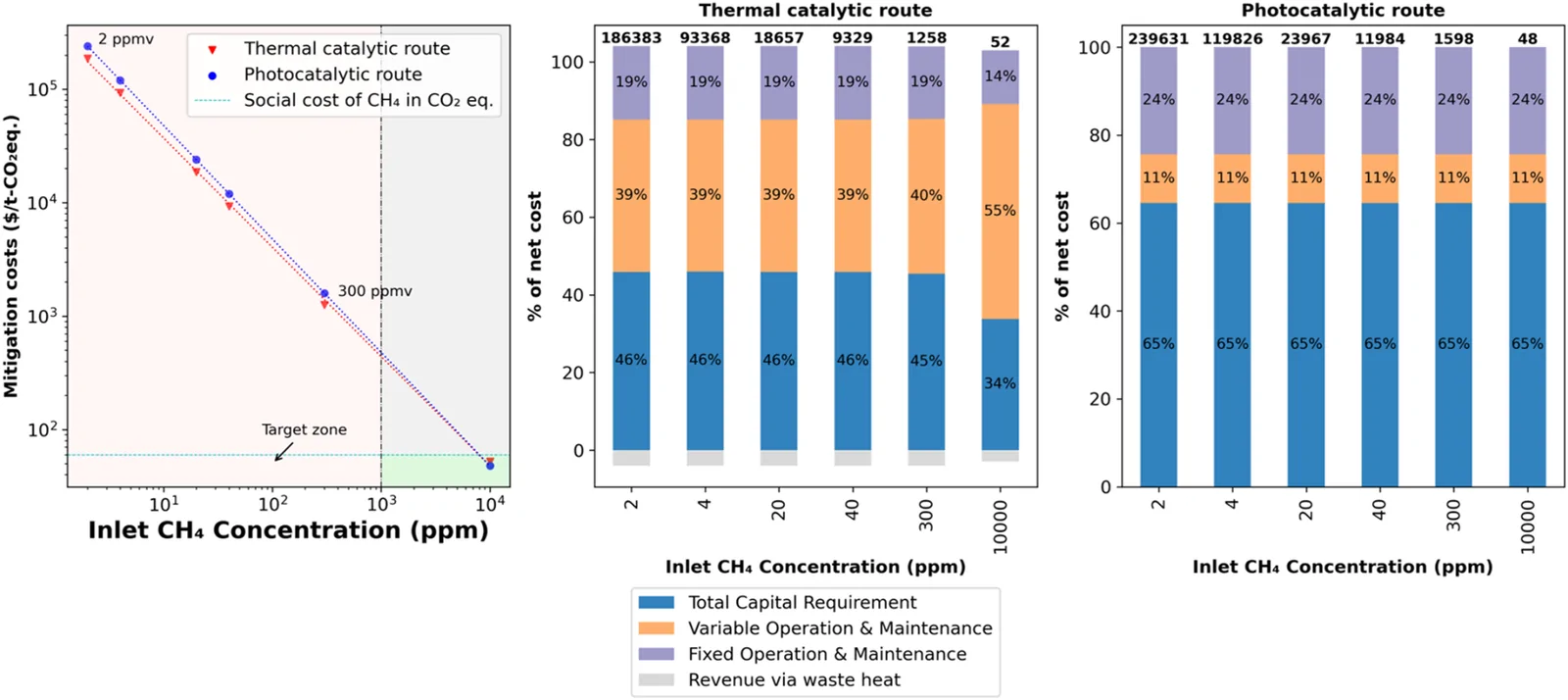
Effect of inlet methane concentration (ppm) on methane mitigation costs in thermal and photocatalytic routes. The social cost of methane in the leftmost figure, representing the cost to society of emitting 1 ton of methane in a year, can be taken as the target cost for the methane oxidation process. EPA reports a cost of 60 $/tCO_2_eq for SC–CH_4_ for the year 2030 and a near-term rate of 2.5%. The light green region in the figure represents technologically and economically feasible region, i.e., where commercial technologies for methane oxidation are available, and the cost is below SC–CH_4_. The light gray region represents the technologically feasible region where commercial technologies exist, but the cost is above SC–CH_4_. The light rose region indicates the region where commercial technologies for methane oxidation are not available. The region below SC–CH_4_ line (Target Zone) within the light rose region (pointed by the arrow) is where most of the methane oxidation technologies should aim to land, as that is where most of the methane is emitted globally, as reported in Abernathy et al.,^44^ which states that the majority of methane emissions happen at concentrations below 1000 ppm. The concentrations are representative of the cases mentioned above. The figures on the right are stacked plots in percentage of total cost. Methane conversion is 99% and 92.5% was in the thermal and photocatalytic routes, respectively.

In the thermal catalytic route, the capacities of process equipment except that of the reactor are relatively unaffected by the change in inlet methane concentration since the volumetric flow rate is the same in all cases. As a result, the costs of most equipment remained relatively unchanged in all cases. The mass of catalyst needed for a given conversion increases with methane concentration and at a constant volumetric flow rate; this increases the reactor size and the cost of the catalyst in the thermal catalytic route. Therefore, the share of variable operating and maintenance costs (VOMs) (VOM includes the catalyst costs) in the overall mitigation cost increases with the methane concentration. Additional analysis performed for a 5000 ppm inlet methane concentration results in VOM contributing 47% to the overall specific mitigation cost. The increase in the share of catalyst is shown in Supporting Information. In the case of the photocatalytic route, an idealized plug flow with first-order kinetics for methane oxidation was assumed. The increase in concentration is canceled out by the increased reaction rate for a first-order reaction under idealized plug flow with a constant volumetric flow and conversion. This makes the mass of catalysts independent of the inlet methane concentration for a constant volumetric flow rate and conversion. As a result, the reactor size and the mass of catalysts are constant in the case of photocatalytic (see Supporting Information for the breakdown of costs). A more detailed explanation of the observed trend is present in the Supporting Information.

Methane oxidation to CO_2_ is an exothermic reaction. Therefore, the amount of heat released from the methane oxidation step is dependent on the amount of methane in the inlet air stream. In this study, we assume an isothermal reactor, thereby rejecting any heat released during the oxidation step to the cooling water. We have neglected the heat rejected to the cooling water and the effect of the costs of cooling water use in different cases in the analysis. However, if we consider adiabatic conditions in the thermal catalytic reactor, in the case with 10,000 ppm methane in the inlet air stream, the temperature rise in the stream due to the exothermic oxidation reaction is ∼250 °C. The resulting high-temperature product stream from the reactor can overcome the minimum temperature difference required in the recuperator, eliminating the necessity of it. The cost of methane mitigation decreases from 52 $/t-CO_2_eq for the isothermal reactor conditions to 31 $/t-CO_2_eq for the adiabatic reactor conditions. The results of specific mitigation cost at different inlet concentrations and their breakdown into bare erected cost and operations and maintenance are presented in the Supporting Information.

The social cost of methane (SC–CH_4_) can be a useful starting target for the methane oxidation processes presented in this study. The social cost of a GHG represents the cost burden on society posed by emitting one ton of that GHG in a year. The value for SC–CH_4_ was obtained from the latest EPA report on the social cost of GHGs.^43^ The social cost of methane for the year 2030 and near-term rate of 2.5% is 1900 $/ton CH_4_.^43^ The social cost of methane oxidation is the social cost of methane minus 2.75 times the social cost of CO_2_.^44^ Since we present methane oxidation in this study, the social cost of methane oxidation would therefore be 1515 $/ton CH_4_. One ton of CH_4_ (equivalent to 28 tons of CO_2_) oxidation produces 2.75 tons of CO_2_; therefore, the derived social cost of methane oxidation is for 25.25 tons of CO_2_. Thus, the social cost of methane oxidation in t-CO_2_eq is $60. Except for the emission scenario representing manure storage, the cost of methane mitigation remains orders of magnitude higher than the social cost of methane.

### 3.3 Sensitivity Analyses

This section presents sensitivity analyses of key process variables and parameters for the overall cost of methane mitigation. The variables to perform sensitivity analysis were considered based on their relevance to the study and the cost reduction potential. Some of the sensitivity studies are specific to either thermal or photocatalytic routes because of the relevance of a variable to that particular route. All of the other remaining design conditions in the process were kept constant and are the same as the reference case.

Variation with methane conversion, one of the key variables influencing both the specific energy demand and the cost, is presented in Section 3.3.1. The effect of catalyst lifetime on specific cost of mitigation is presented in Section 3.3.2. Sensitivity studies for a hypothetical process without a dehumidifier are presented in Section 3.3.3. The reason to consider sensitivity for cases without a dehumidifier is 2-fold: the dehumidification unit consists of several pieces of equipment and, when considered together, has the largest capital share in the thermal catalytic route and the second-largest in the photocatalytic route. Further, it contributes to about 30% of the total energy demand in the thermal catalytic route and 50% in the photocatalytic route. Finally, as seen in Table 5, the gas–gas recuperating heat exchanger is a major cost component in capital expenses in the thermal catalytic route. This is primarily due to the low overall heat transfer coefficient associated with gas–gas heat exchange. The required heat transfer surface area also varies because of the temperature difference between the two streams in the recuperator. Sensitivity with respect to the minimum temperature difference in the recuperator for the thermal catalytic route is included in the Supporting Information. A table containing the summary of sensitivity analyses is presented in the Supporting Information (Table S1).

#### 3.3.1 Effect on Methane Mitigation Costs With Respect to Methane Conversion in the Reactor

Figure 4 shows the sensitivity results with methane conversion varied from the reference case (99% for thermal catalytic and 92.5% for photocatalytic route) value to 20% for both the thermal- and photocatalytic routes. The mitigation costs decrease with conversion up to a value before the costs start to increase again. The effect was less pronounced in the thermal catalytic route, where the lowest mitigation cost was observed for the 90% conversion case. However, for the photocatalytic route, the lowest mitigation cost was observed for the 80% conversion case. Designing the process for lower conversion will require a lower amount of catalyst in both routes. This will also reduce the reactor costs, as a smaller reactor is enough to hold a lower amount of catalyst. However, in both routes, the number of CO_2_ equivalent mitigations decreases with conversion. This, in turn, would increase the mitigation costs. The interplay between these opposing factors determines the net increase or decrease in the cost with conversion.

**Figure 4:**
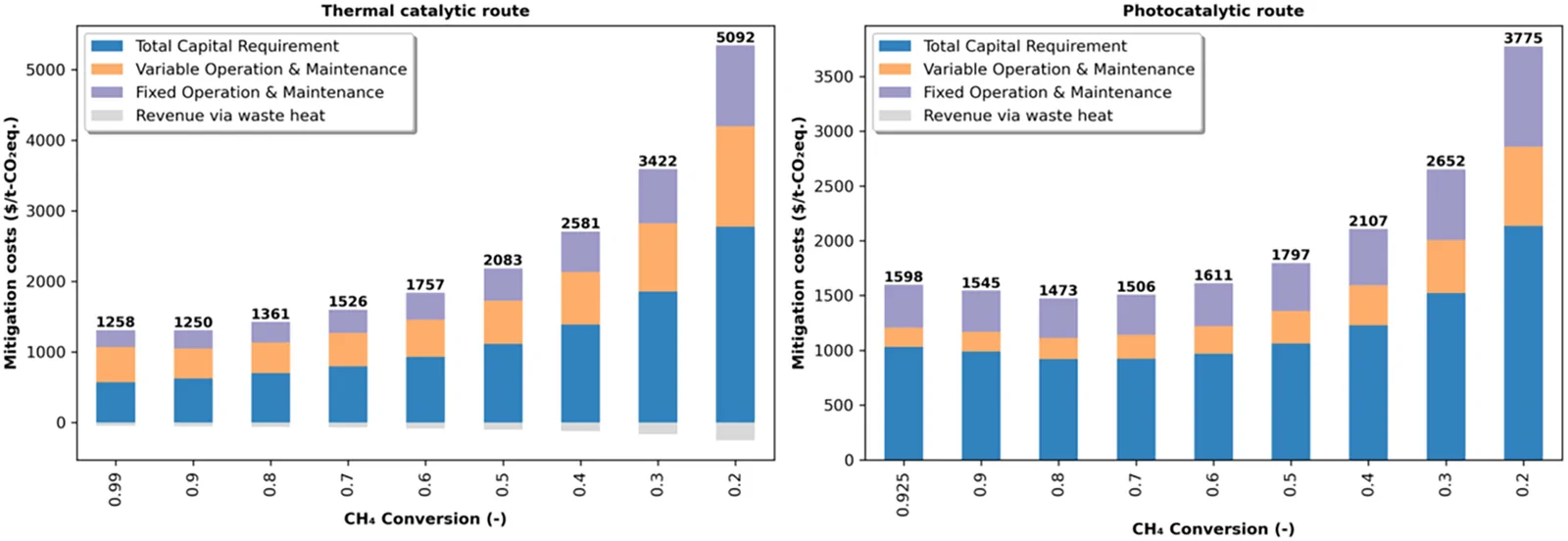
Sensitivity with methane conversion in both thermal- and photocatalytic routes.

Figure 5 captures the effect on migration costs as a function of the conversion level. The amount of catalyst is kept the same, and the conversion of methane is varied. The sensitivity captures the varying effects of the concentration and conditions on conversion for the same amount of catalyst. Clearly, the mitigation costs are higher as the methane conversion decreases. Breakdown of the total cost into capital and operating expenses is presented in the Supporting Information. It should be noted that the analysis assumes complete oxidation of methane to form CO_2_. However, if other partially oxidized products are formed, their yields and any downstream treatment requirements could adversely affect both the mitigation potential and the economics.

**Figure 5:**
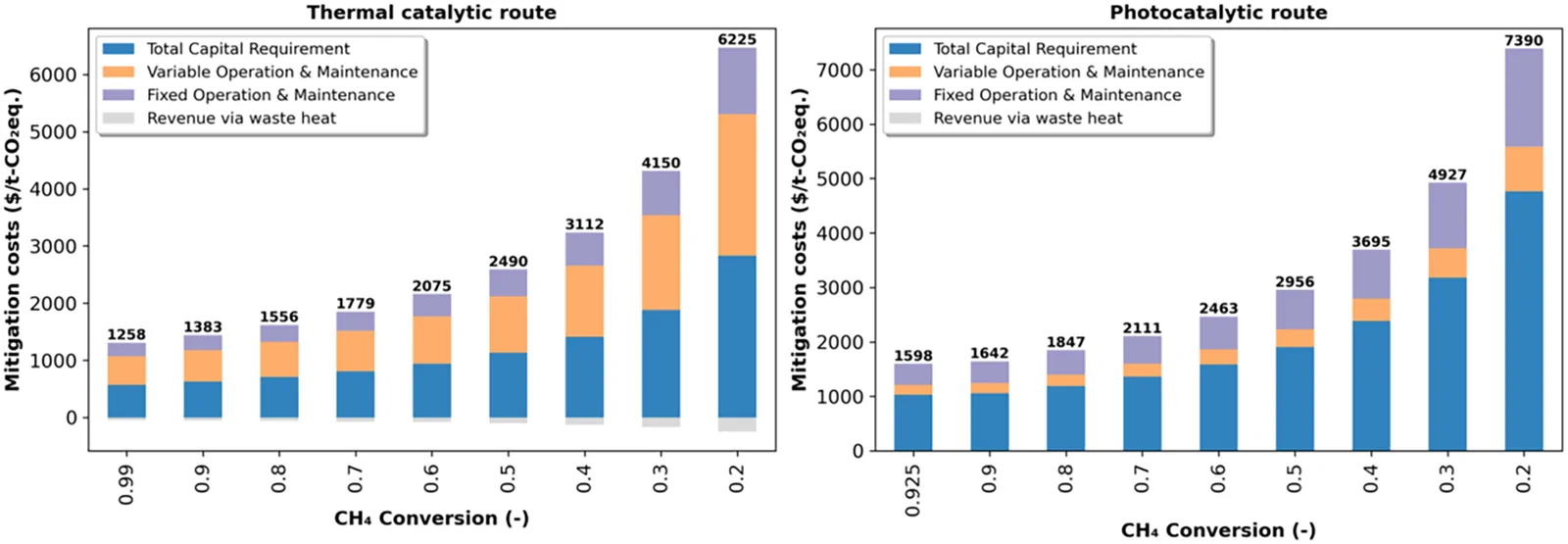
Sensitivity of methane conversion in thermal and photocatalytic routes with the same catalyst amount.

#### 3.3.2 Effect on Costs of Methane Mitigation With Respect to Catalyst Lifetime

The sensitivity of mitigation cost to catalyst lifetime in both the thermal and photocatalytic routes is shown in Figure 6. Catalyst lifetime is influenced by several factors, including the presence of poisonous gases in the inlet stream. In the current study, we only considered the inhibiting effect of moisture and ignored the effect of other gases. A catalyst lifetime of 20 years is considered in the reference case for both the thermal and the photocatalytic routes. The mitigation cost increased by 54% and 2% when the catalyst lifetime is decreased from 20 to 5 years in the thermal and photocatalytic routes, respectively. The decrease is not significant in the photocatalytic route due to its lower cost of the catalyst. The breakdown of different cost components for different catalyst lifetimes is shown in the Supporting Information.

**Figure 6:**
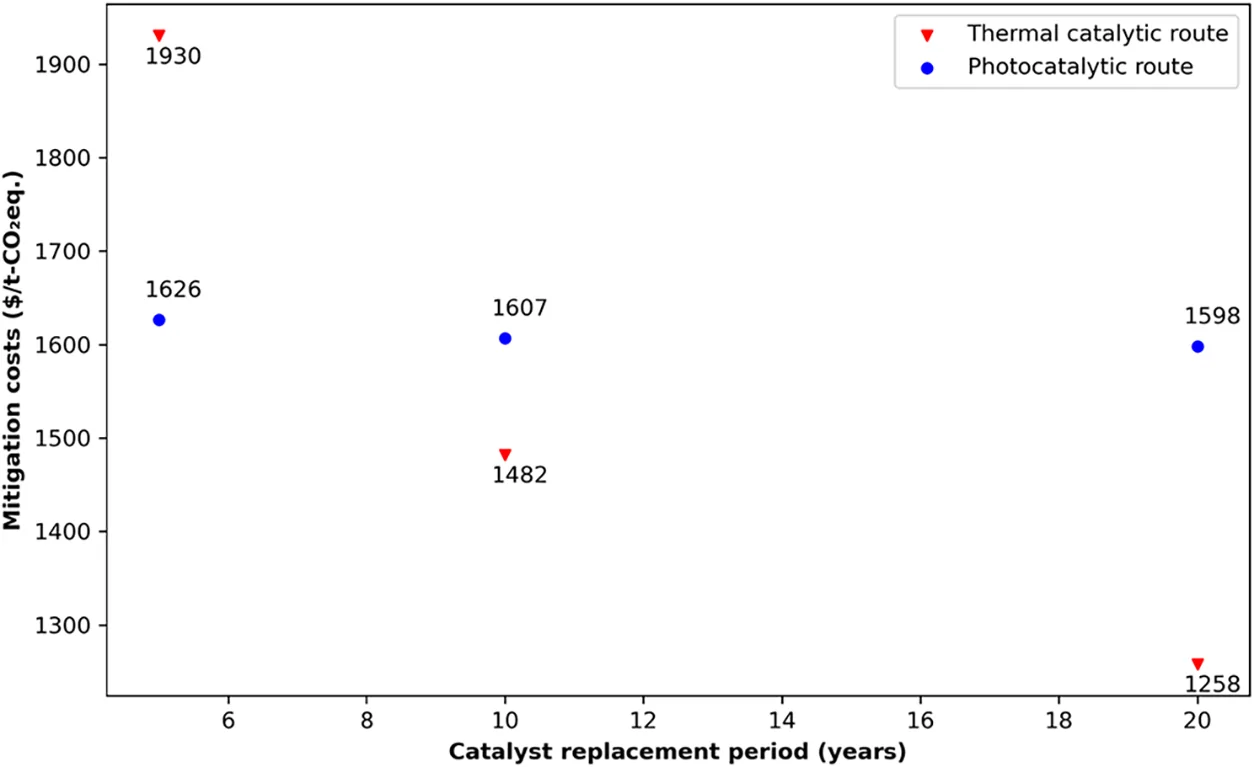
Sensitivity with catalyst replacement in thermal and photocatalytic routes.

#### 3.3.3 Effect of Methane Conversion and Inlet Methane Concentration for the Process without Dehumidifier

The dehumidifier lowers moisture content in the stream entering the reactor (see Figure 1 for process schematic). As briefed in Section 2 on the thermal catalytic route, the dehumidifier is crucial to the catalyst (Pd on Al_2_O_3_) considered in this study owing to its sensitivity to moisture. The variation in the amount of catalyst at different relative humidity (RH) levels can be estimated using the relation presented in Alyani et al.^21^ The relation (presented in Section 2) was used to estimate the change in the amount of catalyst and, as a result, the size of the reactor. The sensitivity includes the effect of higher RH on catalyst mass and related costs. Therefore, the costs are estimated in the thermal catalytic route by including the inhibitory effect of higher RH in a process without a dehumidifier. However, it can also be implied that the thermal catalytic route will have lower overall cost simply due to the absence of a dehumidifier and its associated costs. Therefore, the costs in this sensitivity for the thermal route reflect the trade-off between the performance loss of the catalyst (captured by using more catalyst mass) and using a dehumidifier. Additionally, in the absence of a dehumidifier, the temperature of the cold stream inlet to the recuperator is 34 °C, which is less than in the case that includes the dehumidification step. With a minimum temperature approach of 20 °C in the recuperator, there is not enough sensible heat left in the stream coming out of the reactor that can be repurposed (for example, in a district heating network). Therefore, the revenue generation through waste heat supply is absent in cases without a dehumidifier.

Figure 7 presents the costs per tonne CO_2_-eq mitigated for different inlet methane concentrations and conversion in the reactor for the process without the dehumidification step. For the reference case with 300 ppm inlet methane concentration and in the absence of the dehumidification step, the cost of methane mitigation is 1123 $/t-CO_2_eq and 1024 $/t-CO_2_eq in the thermal and photocatalytic routes, respectively. This is 11% and 36% lower when compared to the case with a dehumidification step in the thermal and photocatalytic routes. Results show that the specific cost in the thermal catalytic route is between 48 $/t-CO_2_eq and ∼168,000 $/t-CO_2_eq for the inlet methane concentrations between 10,000 and 2 ppm (as in ambient air). This is approximately 8% and 10% lower than the case with a dehumidifier for scenarios with 10,000 and 2 ppm inlet methane concentrations, respectively. In the case of the photocatalytic route without a dehumidification step, the costs are between 31 and ∼154,000 $/t-CO_2_eq for the inlet methane concentrations between 10,000 and 2 ppm. The costs without a dehumidifier are approximately 35% and 36% lower than the process with dehumidifier for 10,000 and 2 ppm concentrations, respectively.

**Figure 7:**
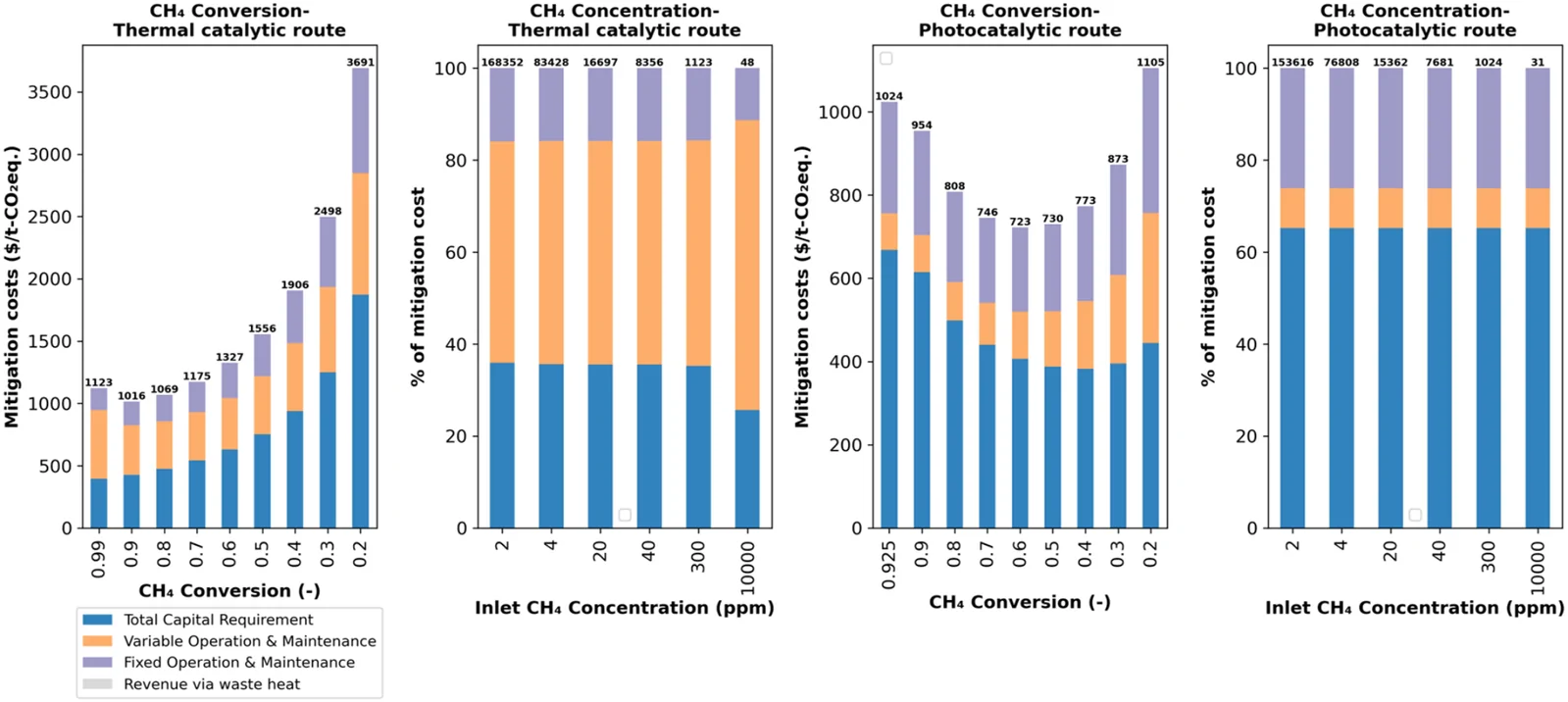
Sensitivity of specific cost for the cases without dehumidifier in thermal and photocatalytic routes. Sensitivity plots with concentration are shown as a percentage of total cost.

The cost decreases with concentration in a similar trend as observed in the case with a dehumidifier. With regard to methane conversion, the costs initially decreased, followed by an increase at lower conversion. The observed trend reflects the net result of two competing factors: the decrease of reactor and catalyst costs with conversion and an increase of costs due to the lower amount of CO_2_ equivalents mitigated with conversion.

The effect of RH or moisture on the thermal catalyst has been presented in the literature, but the effect on the photocatalyst system is not clearly defined. Therefore, unlike the costs calculated for the thermal catalytic route, the costs in the photocatalytic route without the dehumidifier do not consider the higher catalyst mass to reflect the loss in catalytic activity because of moisture. The breakdown of different cost components for the case without a dehumidifier is presented in Supporting Information.

The current study limits the effect of pollutants on the catalyst activity to H_2_O. The presence of other gases in the inlet stream might also have a negative effect on the catalytic activity. For example, Pd-based catalysts were shown to have reduced thermal catalytic activity in the presence of both H_2_S and SO_2_.^45^ Incorporating such pollutant removal methods will result in an increase in methane mitigation costs. Similarly, for the photocatalyst (CuO/ZnO), the effect of inhibiting gases was limited to H_2_O, although the reference text^25^ for photocatalysts did not specify the effect of H_2_O on the catalyst. However, a recent study from Kessler et al.^46^ shows that in the presence of moisture, the rate of photocatalytic methane oxidation was reduced for ZnO and ZnO-Ag due to the formation of a water layer around the catalyst. Because of the low solubility of CH_4_ in the water layer around the photocatalyst, the access of photogenerated active species to methane was reduced.

#### 3.3.4 Effect of Reaction Rates and Surface Area of the Catalyst

Catalysts account for 17% and 0.5%, and the reactor accounts for 1% and 15% of the total mitigation costs in the thermal- and photocatalytic routes, respectively.

In this study, Pd/Al_2_O_3_ is the catalyst assumed in the thermal catalytic route, which has a reaction rate of 4.6 × 10^–6^ mol/(g_cat_ s). A more active catalyst (with a higher reaction rate per mole of active sites) would require fewer active sites (catalyst mass) for the same reaction conditions, assuming no mass transfer limitations. This would reduce the costs related to catalysts and the reactor. Further, the use of cheaper catalysts, for example, other metal oxides like cobalt and iron oxides, will reduce the overall mitigation costs.^47^ This is, however, conditional on those catalysts having a similar or higher catalytic activity at the temperature considered in this study, among other characteristics like inhibition. In this section, techno-economic performance based on potentially highly active catalysts for methane oxidation is presented. In the case of the thermal catalytic route, considering alternative catalysts that have higher reaction rates can bring down the mitigation costs significantly. For example, considering bimetallic catalysts based on Pd on halloysite nanotube supports reported by Tsopelakou et al.^48^ can reduce the mitigation costs by ∼250 $/t-CO_2_eq (∼20%) with respect to the reference case. These catalysts have a maximum reaction rate of 3.65 × 10^–4^ mol/(g_cat_ s), which is ∼80 times the reference case at the reaction conditions under the tested conditions. The decrease in costs is mainly due to the lower amount of catalyst required.

In contrast to the thermal catalytic route, the reactor is the major cost contributor in the photocatalytic route. Using the same CPC setup as described in Section 2.3.2, the cost of photocatalytic reactors can be decreased by reducing the required collector area for an identical conversion. This is achieved through using photocatalysts with a higher activity at the same spectral wavelengths. A sensitivity study with reaction rate is therefore performed to assess the potential decrease in cost using catalysts of higher photoactivity. The range of reaction rate is from 10^–9^ to 10^–3^ mol/(m^2^ s),^48^ while in the reference case the reaction rate was assumed to be 7.3 × 10^–10^ mol/(m^2^ s). Photocatalysts that exhibit the aforementioned range have been tested under a range of light conditions (UV, UV–visible, etc.), but the catalysts operating in the solar spectrum have a slightly higher rate.^48^ It is to be noted that the study from which these reaction rate ranges were obtained also included partial oxidation of methane in their analysis; the catalysts were tested at different spectral ranges, and at higher methane concentrations than considered in this study. Therefore, the sensitivity study is intended to highlight the effect of reaction rate on mitigation costs and to guide screening of catalysts suited for methane mitigation via a photocatalytic route. Since methane conversion also depends on the active surface area of the catalyst, the reaction rates were considered for different surface areas between 32 m^2^/g (for CuO/ZnO in Li et al.^25^) and 50 m^2^/g (for TiO_2_ in Hoffmann et al.^49^).

The sensitivity plot with reaction rate and surface area is shown in Figure 8 for the cases with and without the dehumidifier. In the case with the dehumidifier, the mitigation costs decrease from ∼1600 to ∼740 $/t-CO_2_eq when the reaction rate changes from 10^–9^ to 10^–3^ mol/(m^2^ s). The specific cost decreases and approaches a limit with increasing reaction rate and surface area. The decrease is limited by the lowering of the cost of the reactor and catalyst, while the cost of the rest of the components remains constant, since the volumetric flow rate of the stream passing through the system is the same. A higher reaction rate needs less catalyst; therefore, the size (number of receiver tubes) of the reactor decreases, which significantly increases superficial velocity in each tube. This is further explained in the Supporting Information. If the reactor is sized based on the reaction rate, the specific cost for the cases with and without a dehumidifier approaches ∼740 $/t-CO_2_eq and 165 $/t-CO_2_eq, respectively, at a reaction rate of 10^–3^ mol/m^2^s.

**Figure 8:**
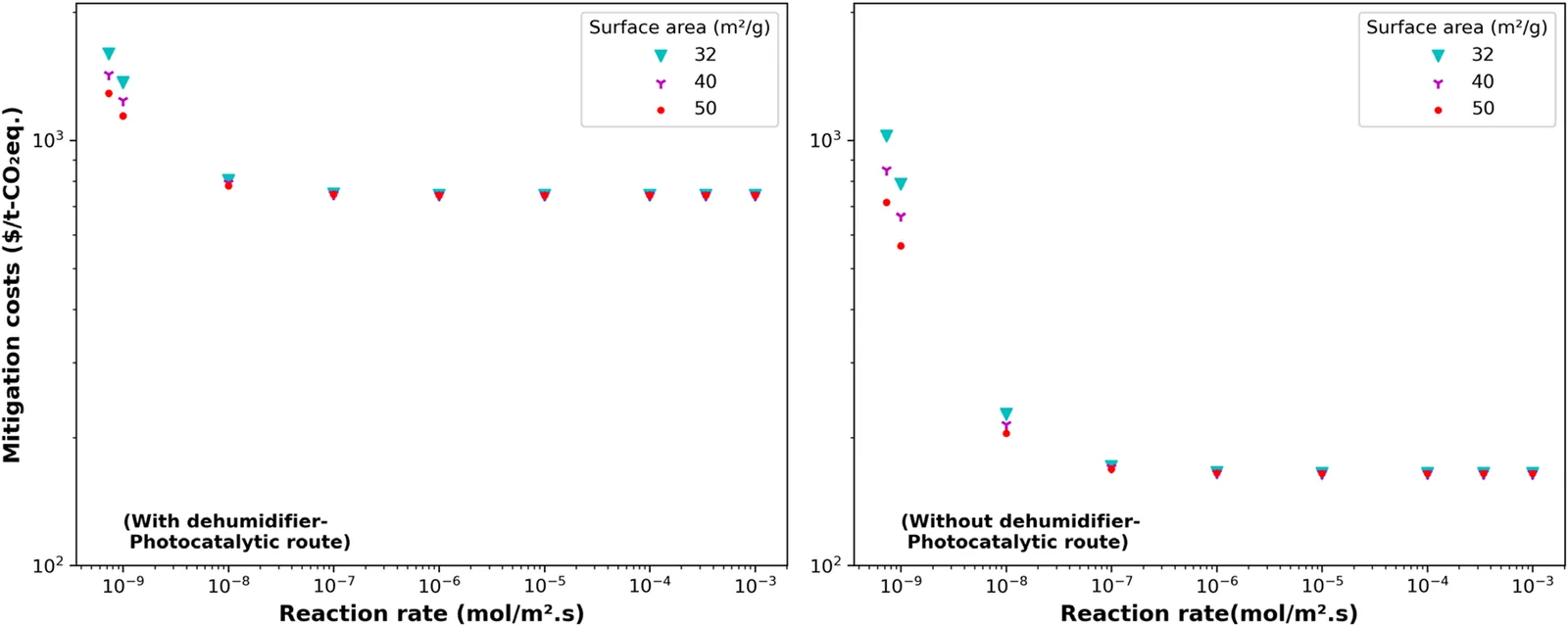
Sensitivity analysis of the specific cost of methane mitigation in the photocatalytic route with reaction rate and the surface area of the catalyst.

It can also be seen in Figure 8 that at higher reaction rates, the difference in mitigation cost is negligible with a change in surface area between 32 and 50 m^2^/g catalyst. The sensitivity study highlights that the methane mitigation costs from the photocatalytic route could reach below 200 $/t-CO_2_eq using a photocatalyst that is uninhibited by water and highly active under conditions resembling natural solar irradiation.

#### 3.3.5 Effect of Assumed GWP on Mitigation Costs

Since methane is a short-lived gas, with a lifetime of 12 years^16^ in the atmosphere, its GWP value is lower if longer timeframes are considered. In this study, methane mitigation is reported in CO_2_ equivalents using GWP100 of 28.^50^ However, if we consider different timeframes and the related GWP, the mitigation costs change significantly. A sensitivity study on mitigation costs with GWP 20 (84, according to AR5 ^50^) and GWP 100 for thermal and photocatalytic routes is presented in Figure 9. Figure 8 also compares the methane mitigation costs against the costs for the direct air capture of CO_2_ process (using the solid sorbent technology).^51,52^ The higher the assumed GWP value, the lower the methane mitigation costs are. It also implies that for near-term climate targets, methane mitigation will be more cost-effective than longer-term climate targets when compared to mitigating or removing CO_2_ from the atmosphere. In a way, if the policy targets near-term warming reduction to prevent midcentury temperature overshoots, thereby avoiding irreversible tipping points, then the measures targeting such emissions, for example, methane, should be evaluated using a metric such as GWP20, which considers its near-term warming impacts. Methane mitigation can be cost-competitive against existing direct capture of CO_2_ technologies considering GWP20 and for conditions having higher inlet methane concentration. The costs for methane mitigation via the thermal catalytic route are below 200 $/t-CO_2_eq when the inlet methane concentration is ∼2300 ppm or higher in the inlet stream, assuming GWP100 as 28. Therefore, for streams having lower concentrations, an energy- and cost-effective upgrading step can make it competitive when compared to state-of-the-art technologies that can capture CO_2_ from ambient air.

**Figure 9:**
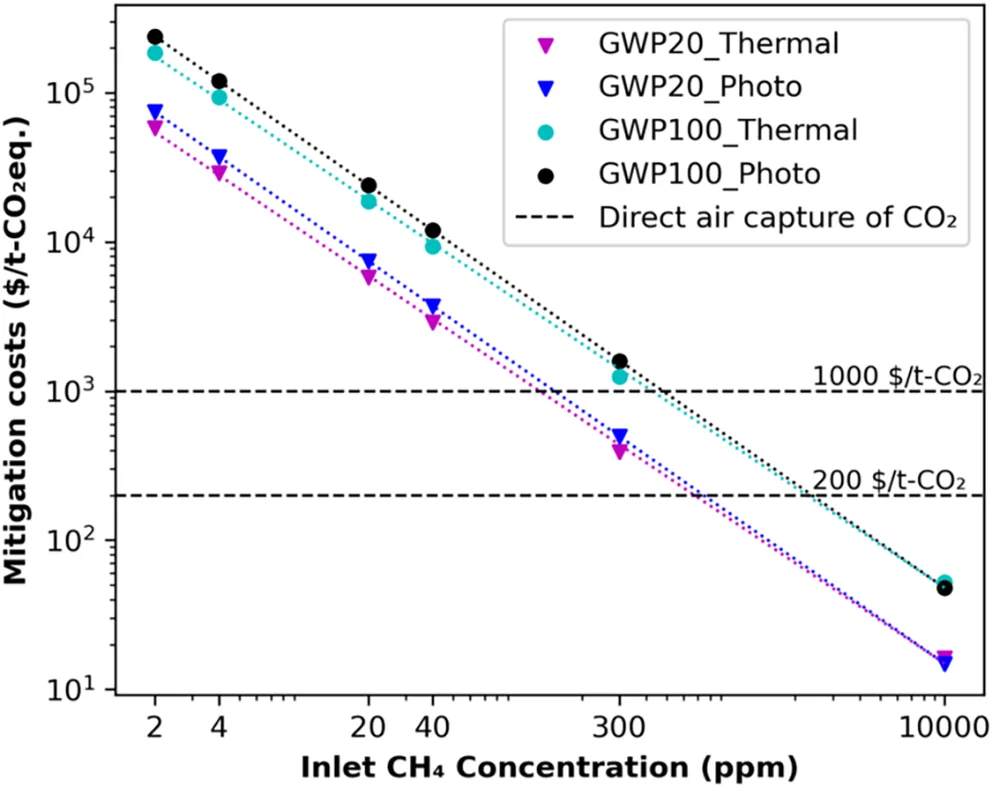
Sensitivity of total cost in thermal catalytic route with global warming potential.

#### 3.3.6 Effect of Emissions Intensity of the Energy Source on Mitigation Costs

The analysis presented in the previous sections does not consider the emission intensity of the source of energy. Considering these emissions is important, as the cost—which evaluates cost per ton CO_2_ mitigated—is dependent on CO_2_ emissions even from the energy source. All the energy demand in the process is assumed to be satisfied using electricity. Using the emission factor for the Nordic consumption mix as 93.2 gCO2eq/kWh,^53^ the cost in the thermal catalytic route for the reference case increased by 68% to 2108 $/tCO2eq. For the European mix with an emission factor of 293 gCO_2_eq/kWh, there is a net release of CO_2_ emissions from the process, i.e., emissions from energy demand are higher than the emissions mitigated by the process through oxidation. The results suggest the importance of using renewable electricity with low carbon intensity to improve the feasibility of the methane mitigation options.

## 4 Conclusions and Implications

This work provides insight into different process-level characteristics of methane oxidation routes that affect their techno-economic performance. The study lays the foundation for different process improvements that should be made to bring down the costs of methane mitigation. In addition, it also highlights the scenarios and conditions under which methane mitigation via oxidation can be applicable. Methane mitigation costs are highly sensitive to the inlet methane concentrations, hence impacting both the relevance and cost of these technologies. Methane oxidation routes can economically compete with direct air capture of CO_2_ for scenarios with methane concentrations higher than 300 ppm. By choosing 300 ppm, we presented the techno-economic feasibility of the best case for that source, at least based on concentration, showing the potential for the technology under optimal conditions. With further developments in catalysts and process design, the oxidation routes can become economically viable even for scenarios with methane concentrations of less than 300 ppm. Directly oxidizing natural methane emissions present in ambient air or from wetlands is highly unlikely to be implemented because of the very high costs. Among anthropogenic sources of methane emissions, such technologies can be especially relevant for the agricultural sector. While the technologies could fit as is in the agricultural sector, a preconcentration step would likely be required to make it an even more cost-competitive solution.

In the thermal catalytic methane oxidation route, the costs related to electricity (mainly due to the dehumidification step and preheating the stream), catalyst use, and the capital cost of the heat exchanger are major cost contributors. In the current study, a Pd-based catalyst was used in the analysis. Using cheaper alternative catalysts based on metal oxides reduces the methane mitigation costs. In addition, catalysts that are more robust and can withstand the water vapor (relative humidity) content in the gas could eliminate the need for the dehumidification step. This would bring down the mitigation costs in the thermal catalytic route significantly. Lastly, integrating the thermal catalytic conversion route with an upstream preconcentration step can eliminate the need for the heater since oxidation of methane at higher concentrations can overcome the minimum approach temperature in the recuperator. In the case of the photocatalytic route, the availability of solar irradiance and the catalytic activity affect the mitigation costs. Therefore, regions with high solar potential favor the use of photocatalytic routes. In addition, using catalysts with higher catalytic activity will result in a smaller reactor size, thereby reducing the mitigation costs for the photocatalytic route.

The source of electricity/energy is critical to determining if these technologies result in negative emissions. The higher the GHG footprint, the higher the mitigation costs. In addition, special attention should be given to the choice of the GWP of methane while reporting the mitigation costs. Whether oxidizing methane to a gas (CO_2_) with a lower GWP results in mitigation or removal of CO_2_ equivalents from the atmosphere depends on the source of methane. It is important to consider methane mitigation or removal separately without using the same frameworks that are applicable to CO_2_ mitigation or removal, since mitigating/removing methane results in reducing/removing CO_2_ equivalents but does not directly reduce or remove CO_2_ physically. The discussion is not within the scope of this study and will be addressed in future work.

## Supplementary Material



## Notes

Process modeling, estimating overall heat transfer coefficient, summary of sensitivity analysis (Table S1), breakdown of total costs in sensitivity analyses (Figures S1–S8), effect on costs of methane mitigation with respect to dT—minimum in the recuperator (Figures S9 and S10), and sensitivity with catalyst—calculations for thermal and photocatalytic routes (Tables S2–S5) (PDF)
