# Supplementary material for: Techno-Economic Assessment for Methane Mitigation through Catalytic Oxidation from Low-Concentration Sources
Source: Ind Eng Chem Res. 2026 Oct 21;65(43):22958–72. doi: 10.1021/acs.iecr.6c02071 (PMC13742898; doi:10.1021/acs.iecr.6c02071)
Supplement: Supplementary file 1 [file ie6c02071_si_001.pdf]

Techno-economic assessment for methane mitigation through catalytic oxidation from low-concentration sources

Supporting Information

Devesh Sathya Sri Sairam Sirigina[a*], Sai Gokul Subraveti[b], Simon Roussanaly[b], Rahul Anantharaman[b], Shareq Mohd Nazir[a]**

[a]Department of Chemical Engineering, KTH Royal Institute of Technology, Stockholm - 11428, Sweden
[b]SINTEF Energy Research, Trondheim, Norway

Corresponding authors – *Devesh Sathya Sri Sairam Sirigina, sirigina@kth.se
**Shareq Mohd Nazir, smnazir@kth.se

Process modelling

In the process concepts proposed by Sirigina et al. [1], a process configuration with co-removal of CH_4 and CO_2 was also presented. Co-removal includes CO_2 capture unit and captures CO_2 present in inlet stream along with the CO_2 produced from methane oxidation. A pressure drop corresponding to 5% at its inlet was considered for the CO_2 capture unit. The pressure rise in blower in the co-removal configuration therefore accounts for this pressure drop in CO_2 capture unit. To reduce the number of modelling cases, the pressure rise in blower for the configuration with methane mitigation also accounts for the pressure drop in the CO_2 capture unit, i.e., the pressure rise by the blower corresponds to the co-removal process. A consequence is that the evaluation of methane mitigation case inadvertently considers a higher capacity blower, corresponding to that of the co-removal case, and the energy demand and costs pertaining to it. As is seen in the results, the costs share of the blower is not significant in relation to other costs for the base case with dehumidifier. Similar to thermal catalytic route, the pressure rise in blower for methane mitigation in photocatalytic route corresponds to that of the co-removal case.

Estimating overall heat transfer coefficient

The overall heat transfer coefficient is estimated using the following relation from Peter et al. [2]:

$\frac{1}{U_i} = \frac{1}{h_i} + \frac{1}{h_{i,d}} + \frac{A_i x_w}{k_w A_{m,w}} + \frac{A_i}{h_o A_o} + \frac{A_i}{h_{o,d} A_o}$ where U_i is the overall heat transfer coefficient with inner surface area of the tube as reference. h_i and h_o in the above equation refer to the heat transfer coefficients inside and outside the surface of the tube; $h_{i,d}$ and $h_{o,d}$ refer to the heat transfer coefficients that account for fouling and dirt accumulation inside and outside the surface. Resistances due to the fouling and dirt accumulation are ignored. A_i and A_o refer to the inner and outer surface area of the tube which are estimated using the corresponding diameters (D_i and D_O). Additionally, x_w refers to the wall thickness and $A_{m,w}$ is the mean surface area of the wall given by $\frac{\pi L\,(D_o - D_i)}{\ln\left(\frac{D_o}{D_i}\right)}$. k_w refers to the thermal conductivity of the material. On substituting the area and mean surface area (and ignoring the dirt and fouling factors) and rearranging, the following equation is used to estimate the overall heat transfer

coefficient for different heat exchangers: $\frac{1}{U_i} = \frac{1}{h_i} + \frac{D_i \ln\left(\frac{D_o}{D_i}\right)}{2k_w} + \frac{D_i}{h_o D_o}$. For the evaporator in dehumidification unit, the values for D_i and D_O as 0.01305 m and 0.01905 m, respectively, and the k_w of SS304 as 16.2 W/(m.K) [3]. The inlet stream after the blower is flowing on shell side and the refrigerant on tube side. The heat transfer coefficient for air is between 80-120 W/ (m^2 K). The value of heat transfer for the ammonia in two-phase region is between 2000-8000 W/ (m^2 K). The heat transfer coefficients around the range were evaluated and the obtained mean value of 138 W/ (m^2 K) is used for simulations. For the condenser tubing in dehumidification unit the inner and outer diameters, D_i and D_O, are 0.0254 m and 0.0194 m, respectively. With the same heat transfer coefficient for air as taken in the evaporator, and the heat transfer coefficient for condensing ammonia is in the range of 2839 – 5678 W/ (m^2.K) [4].

Table S1: Summary of sensitivity analyses. The Italicized value is the value in the reference case.

**Scenario / Sensitivity**	**Range**	**Objective**
Methane Conversion	0.2-*0.99 (Reference case)* [thermal catalytic route]; 0.2-*0.925 (Reference case)* [photocatalytic route]	Simulating real life conditions which involves different conversion rates
Catalyst lifetime	5, 10, *20 years (Reference case)*	Effect of catalyst lifetime on the cost; catalyst lifetime could vary due to different reasons including the presence of poisonous gases
Minimum temperature difference in the recuperator	*20 (Reference case)*, 30, 40, 50 K	Effect of size of the recuperator size on cost; recuperator with a smaller heat transfer area is cheaper but operates at higher effectiveness
Process without dehumidifier	Concentration range representative of emission sources specified in Table 1; conversion range as specified above	Effect of dehumidification on cost; estimates cost with catalysts that are moisture resistant
Reaction rate / Different catalyst	PdNi@Hal, PdCo@Hal, PdCu@Hal, Pd@Hal, *6.5 wt% Pd/Al2O3 (Reference case)* [thermal catalytic route]; Reaction rate range 10^{-9} to 10^{-3} mol/(m^2 s), *7.3×10^{-10} mol/(m^2 s)* (*Reference case*) and surface area - 32, 40, 50 m^2/g [Photocatalytic route]	Effect of different catalyst/reaction rates on the cost
Global warming potential	GWP20 and *GWP100 (Reference case)*	Effect of GWP timeframe on cost evaluation; particularly relevant as the process deals with methane which is a shot-lived GHG
Emissions intensity	*0 (Reference case)*, 93.2 gCO2eq/kWh (Nordic consumption mix), 293 gCO2eq/kWh (European mix)	Estimates net CO_2eq mitigated in the process; provides climate breakeven

Breakdown of total costs in sensitivity analyses

The trend of the results that were present in Figure 3 is linear under a log-log plot. For the photocatalytic route, as reported in the manuscript, the capital and operating cost of processing the inlet stream is almost the same across all the concentrations. However, the number of CO2 equivalents removed for each concentration case is proportional to the inlet stream concentration for the same conversion. Therefore, specific cost is inversely proportional to the concentration. The equation representing cost-concentration relation takes the form: $\mathrm{c} \propto 1/\mathrm{x}$, where *c* is the specific cost and *x* is the concentration. The sensitivity results with concentration approximately fit the trend described by the following equation: $\mathrm{c} = \mathrm{k}/\mathrm{x}$, where k is a constant representing total cost. On a log-log plot, the equation yields a linear trend for the specific cost against concentration with a negative slope, as observed in the first plot of Figure 3. The results also show a similar variation and trend in the case of thermal catalytic route, and follow a similar explanation. There is a slight difference in the thermal catalytic route for the catalyst amount and the reactor size which vary slightly with the concentration.

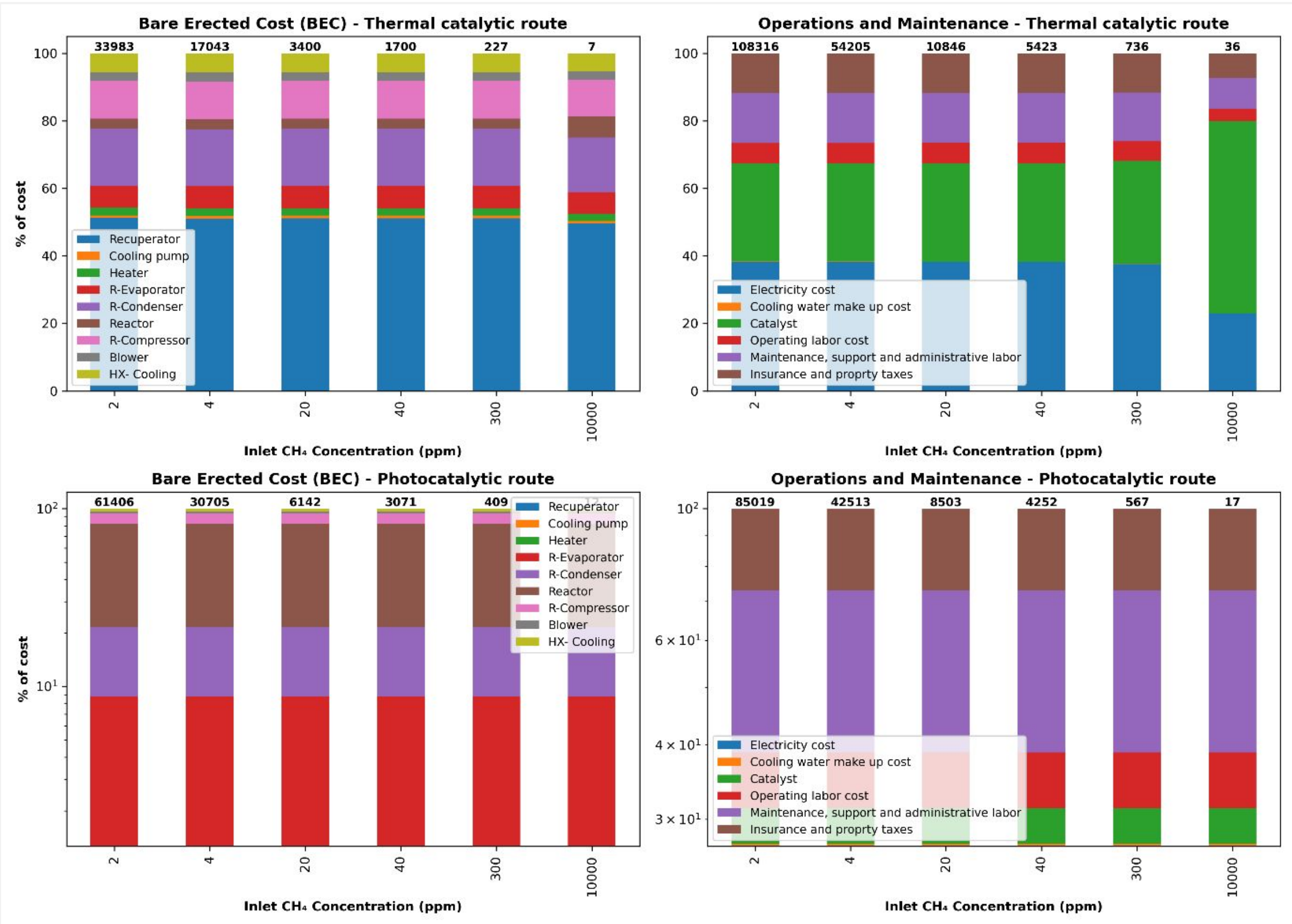

Figure S1: Sensitivity with inlet methane concentration. Breakdown of total costs into capital and operating expenses. The plots are in percentage of costs.

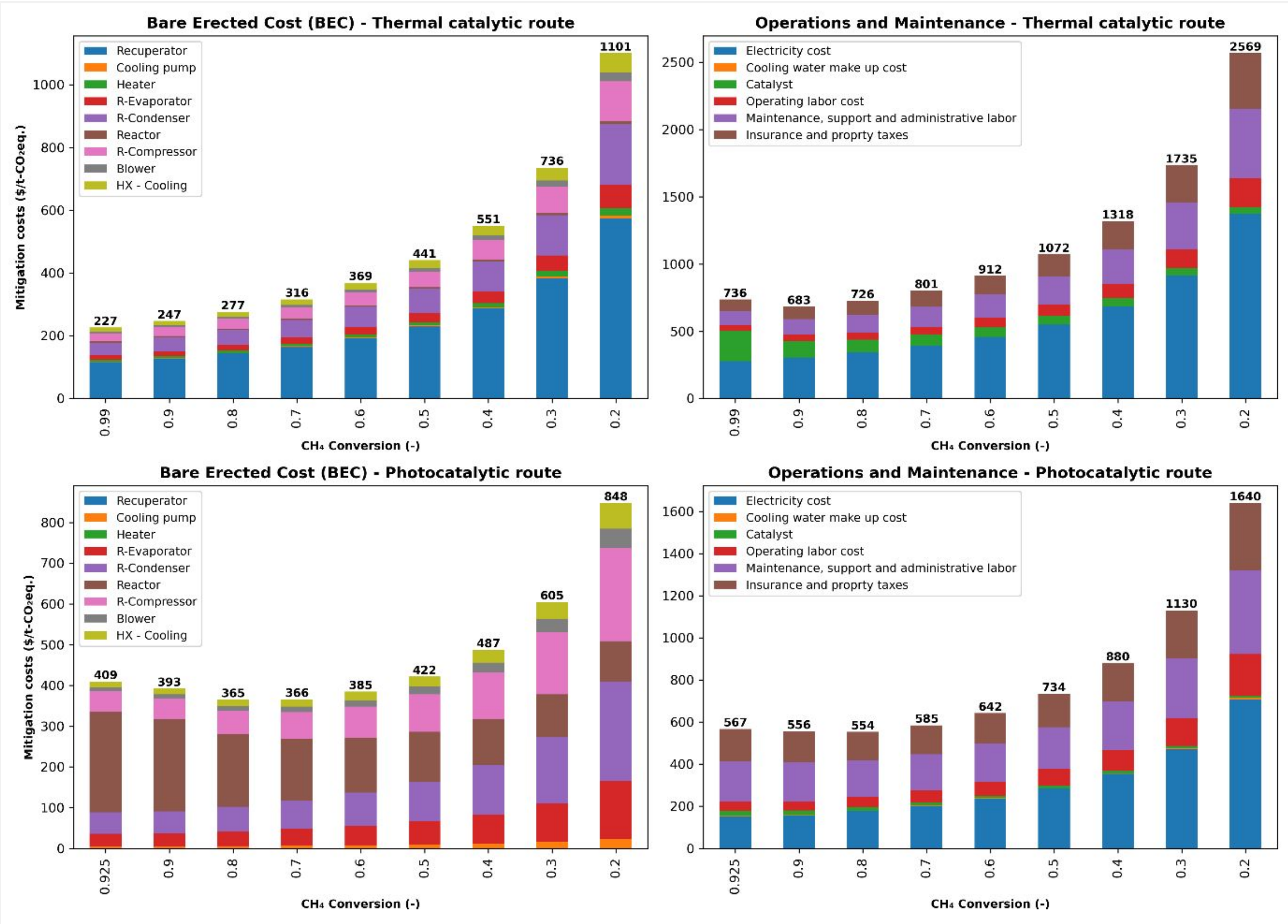

Figure S2: Bare erected cost, and operations and maintenance cost breakdown for sensitivity with methane conversion in thermal- and photocatalytic routes.

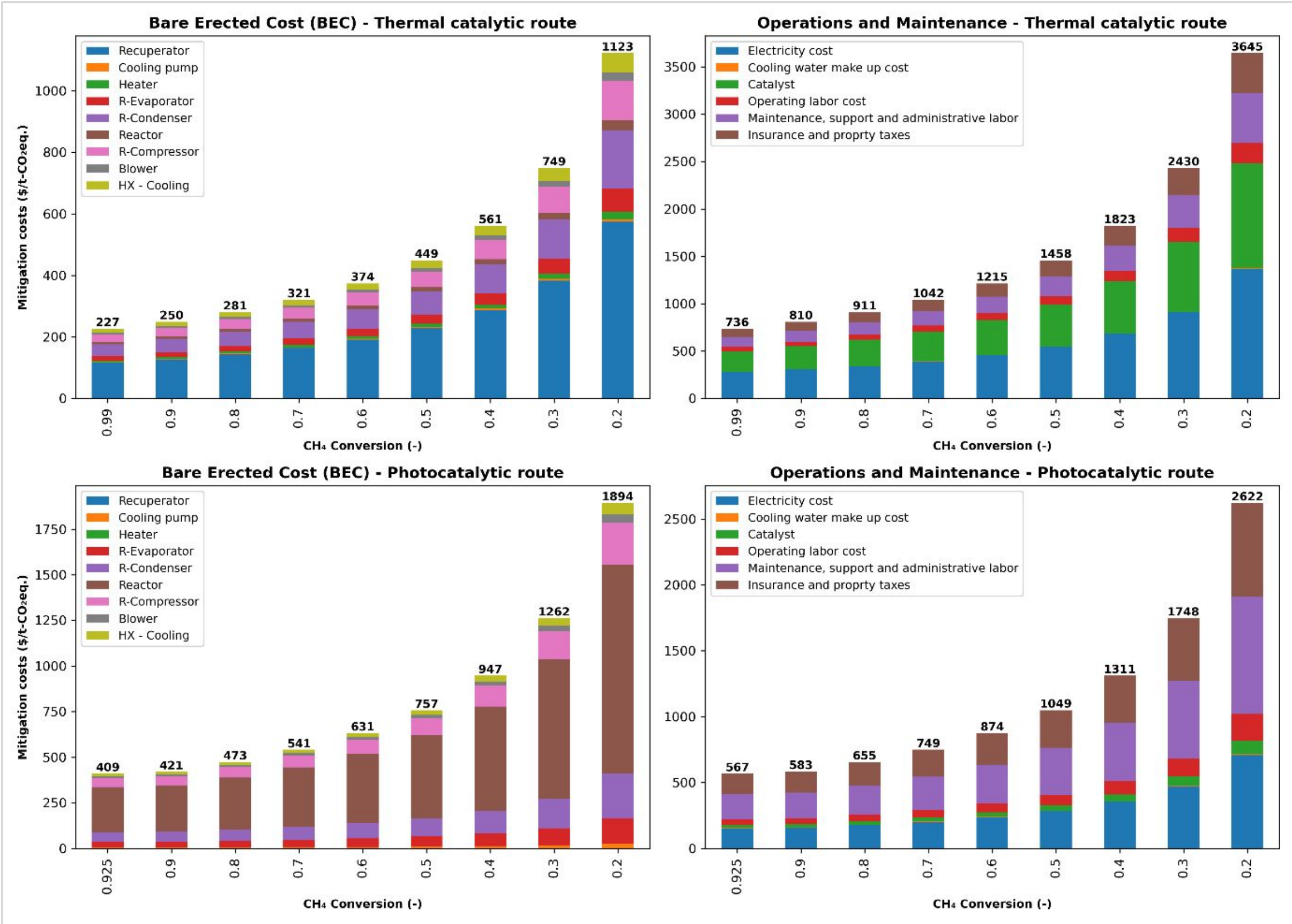

Figure S3: Capital and operating expenses for sensitivity with CH_4 conversion with the same catalyst amount as assumed in the reference case.

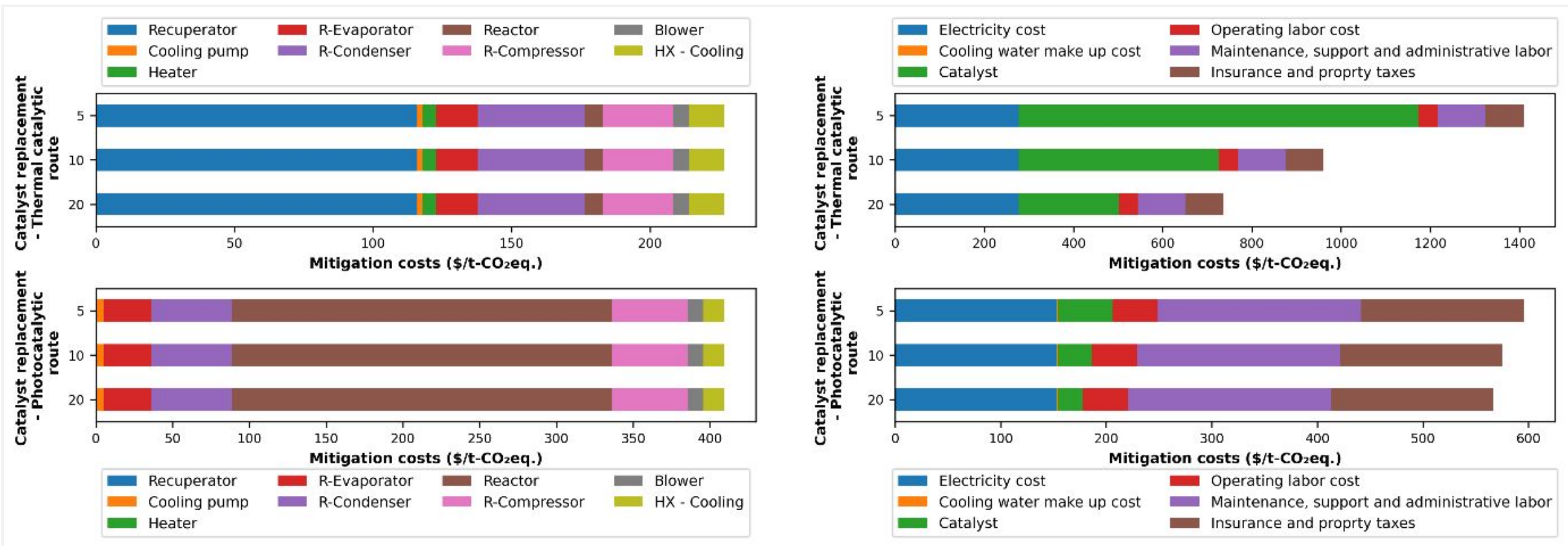

Figure S4: Capital expenses and operating expenses of sensitivity with catalyst replacement in thermal- and photocatalytic routes. The left chart shows capital expenses while the right chart shows the breakdown for operations and maintenance.

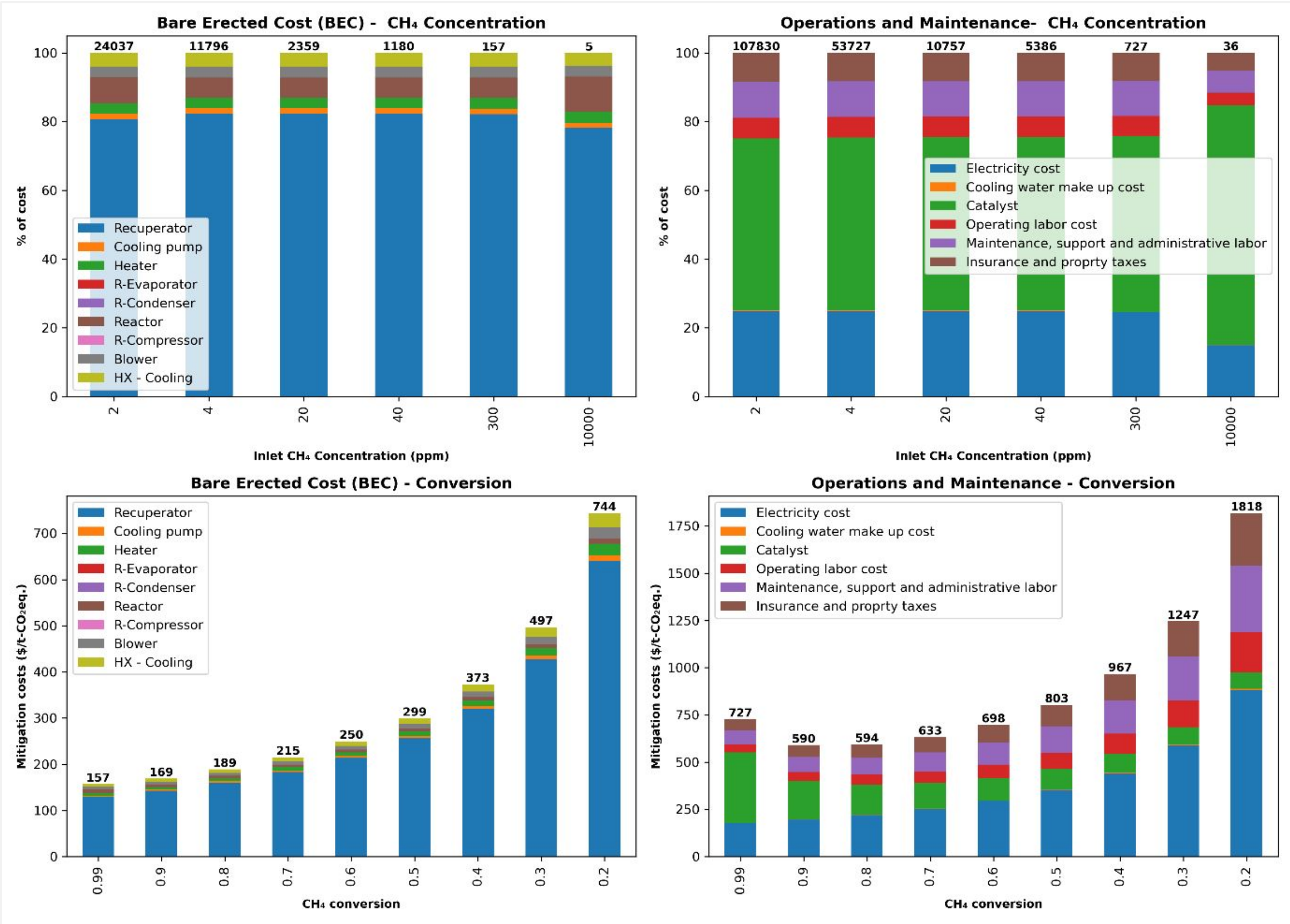

Figure S5: Breakdown of sensitivity without dehumidifier with methane conversion and conversion in thermal catalytic route. Please note that the plots for methane concentration are reported in percentage of costs.

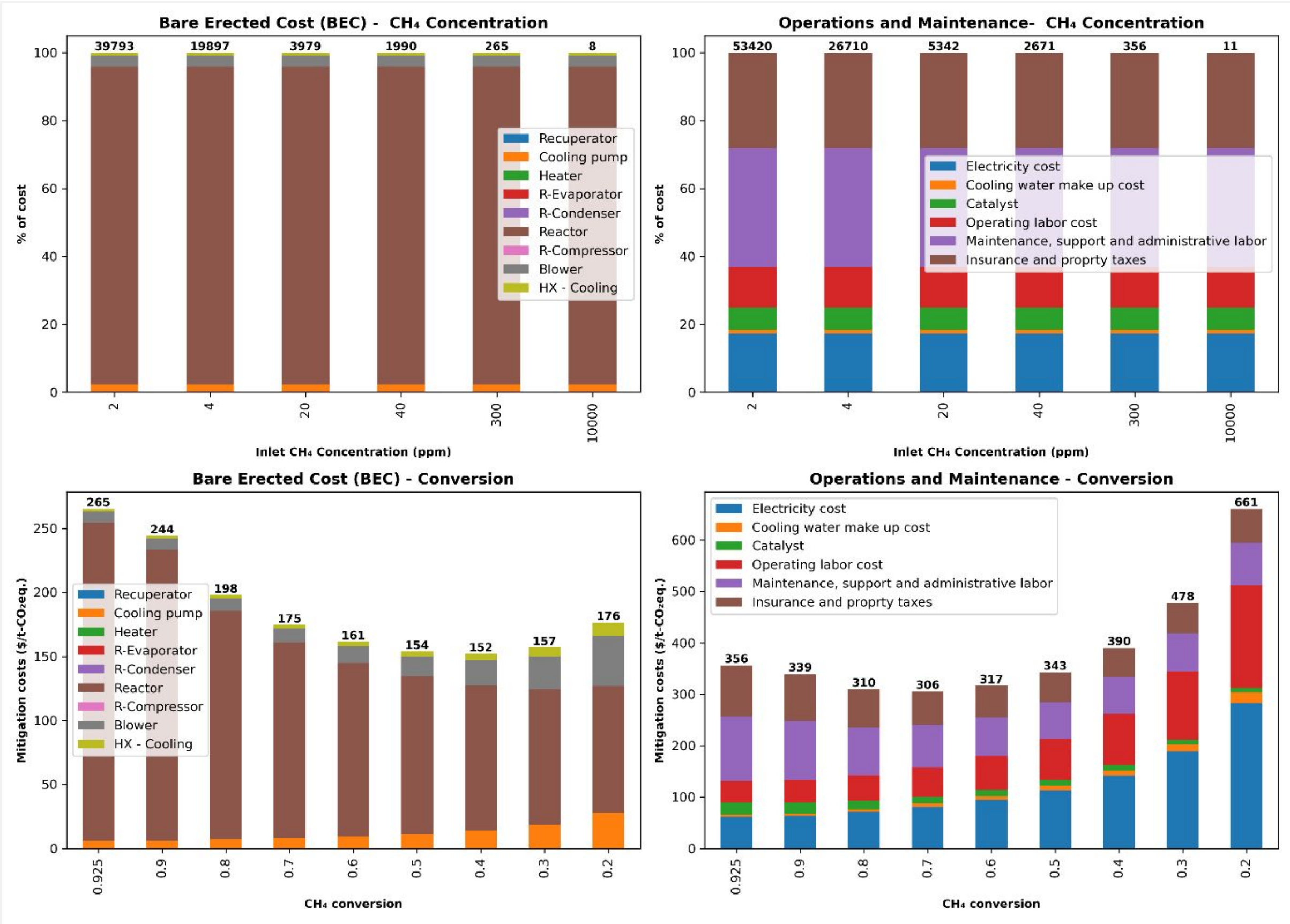

Figure S6: Breakdown of sensitivity without dehumidifier with methane conversion and conversion in photocatalytic route. Please note that the plots for methane concentration are reported in percentage of total cost.

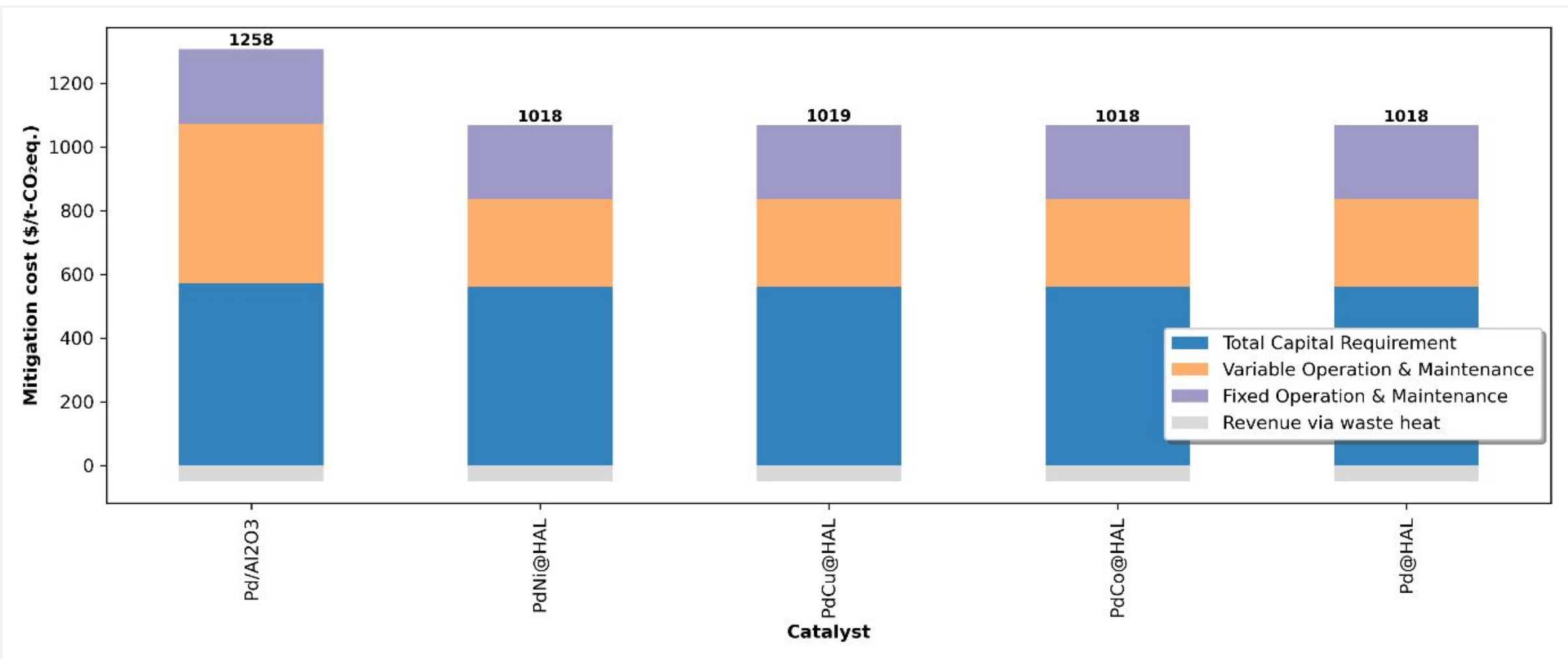

Figure S7: Sensitivity with different catalyst in the thermal catalytic route

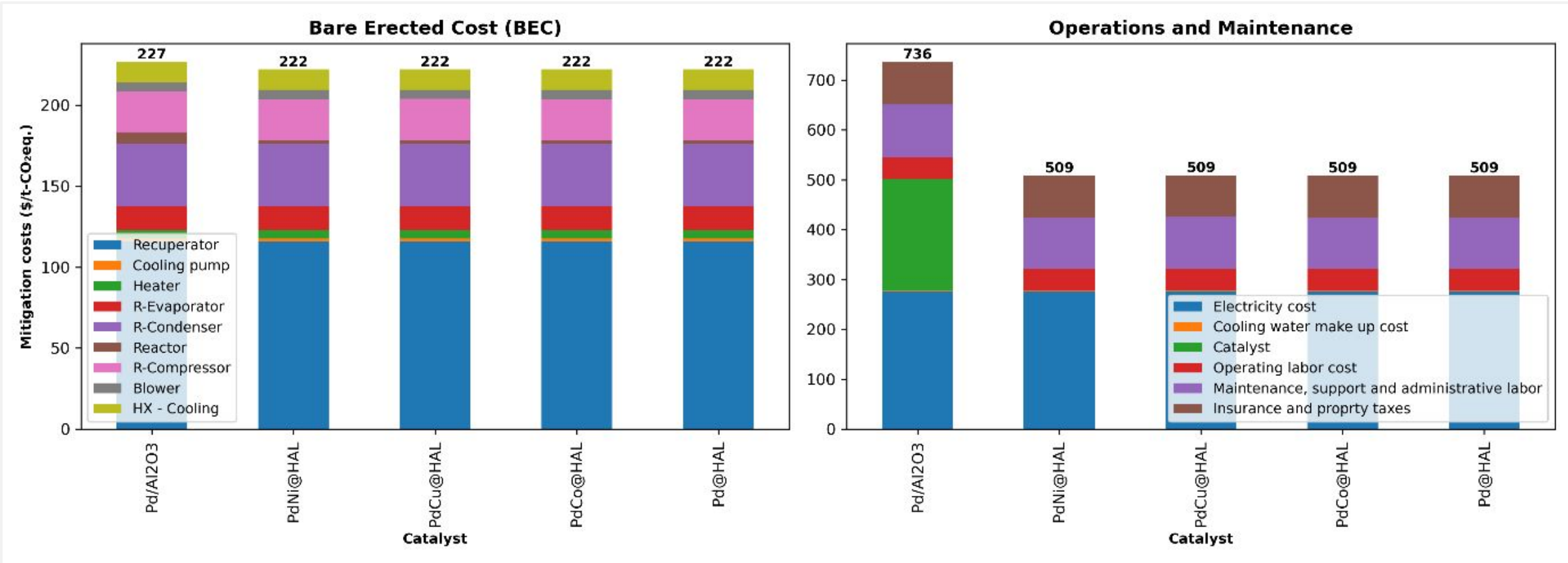

Figure S8: Breakdown of sensitivity with catalyst in thermal catalytic route.

Effect on costs of methane mitigation with respect to dT – minimum in the recuperator

Minimum temperature difference (ΔT_{min}) varies the required heat transfer area and therefore the size and the cost of the recuperator. The sensitivity with ΔT_{min} is shown in Figure S9. A value of 20 K as ΔT_{min} was considered in the base case. The costs decrease with the increase in the minimum temperature difference in the recuperator and seem to approach a limit as the design ΔT_{min} is increased further. This is due to the increase in the costs of heating with the decrease in ΔT_{min} which reduces the temperature inlet of the stream inlet to the process heater. Mitigation cost decreased by 12% from 1258 \$/t-CO_2eq on increasing ΔT_{min} from 20 K to 50 K. This suggests that a recuperator with a higher design ΔT_{min} is a cost-effective option.

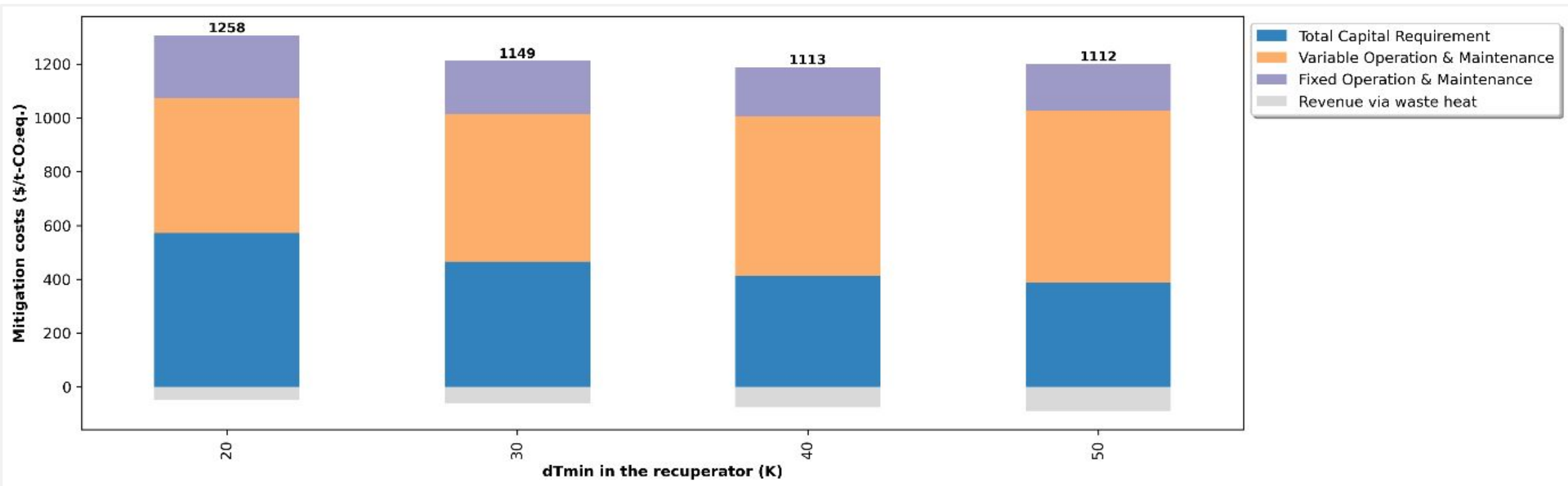

Figure S9: Sensitivity with dT-minimum in the recuperator in thermal catalytic route.

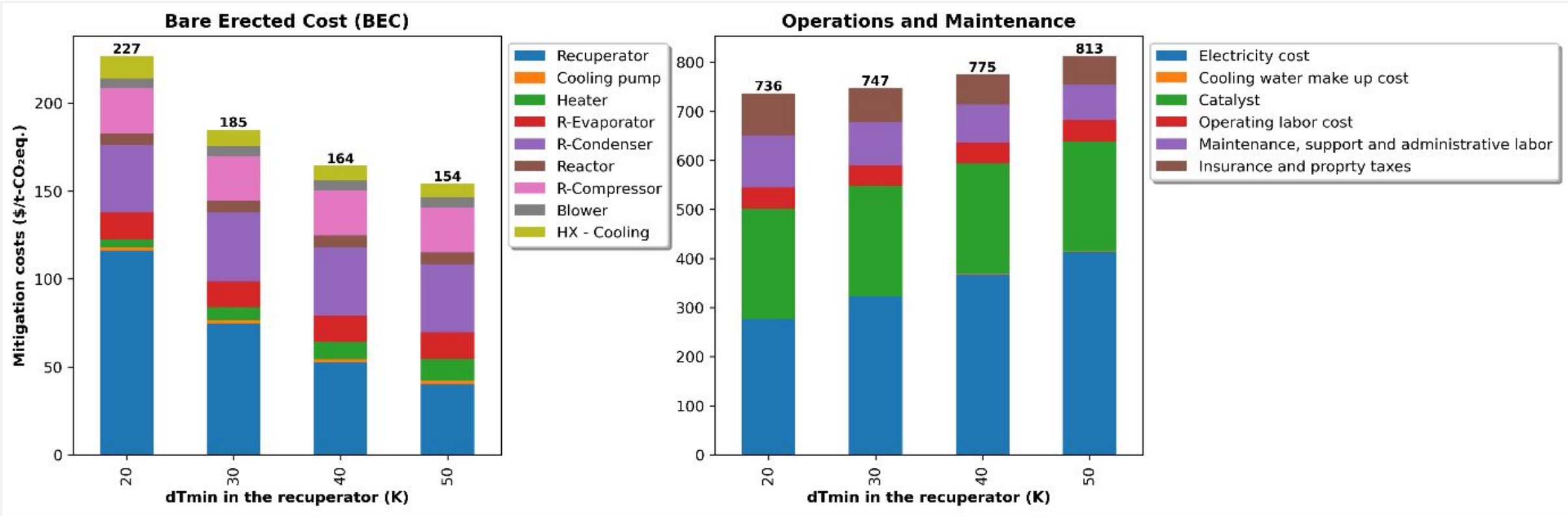

Figure S10: Breakdown of total specific mitigation cost for sensitivity with dT minimum in the recuperator.

Sensitivity with catalyst – calculations

Thermal catalytic route

For each catalyst in thermal catalytic route, the mass required to achieve the conversion of 99% methane in the reactor was estimated. The volume of reactor was estimated using the following plug flow equation:

$$\frac{dF_{CH_4}}{dV} = -r\ (Eq. A)$$

External and internal mass transfer limitations were neglected, and therefore the bulk concentration is assumed to be available at the catalyst site. The rate of reaction for the catalysts PdNi@Hal, PdCo@Hal, PdCu@Hal, Pd@Hal at 250 °C was obtained from [5]. Rate constant from the reaction rate was estimated using the following first order rate equation at CH_4 concentration used in [5].

$$-r = kC_{CH_4} (Eq. B)$$

The reaction rate was reported on mass basis in [5]. It was converted to volumetric basis using following conversion:

$$-r'\left(\frac{mol}{g_{pd}.s}\right) * Pd\_fraction\left(\frac{g_{pd}}{g_{cat}}\right) * \rho_b\ (\frac{g_{cat}}{cc})\ =\ -r\ (\frac{mol}{cc.s})$$

Table S2: Pd composition ($Pd\_fraction$) of catalyst in mass fraction obtained from [5]

**Catalyst**	$Pd\_fraction\ (\frac{g_{pd}}{g_{cat}})$
PdNi@Hal	0.91
PdCo@Hal	0.90
PdCu@Hal	0.94
Pd@Hal	0.92

Table S3: Arrhenius factors of the catalysts estimated from the kinetic data at 250 °C [5]

**Catalyst**	***A***
PdNi@Hal	2.68265E+09

PdCo@Hal	2.86083E+10
PdCu@Hal	2.86439E+11
Pd@Hal	2.76346E+11

ρ_b is 0.65 $\frac{g_{cat}}{cc}$ for all the catalysts. Bulk density of the catalyst in the reactor bed was calculated using the particle density of catalyst and bed porosity. A bed porosity of 0.4 is assumed. Particle density of the composite catalyst is estimated using the weight fraction and solid density of individual elements. Given the porous nature of Halloysite nanotubes (HNTs), the density of HNTs was estimated using its solid density of 2.54 g/cc [6], and a pore volume of 0.54 cc/g [5]. Using the rate, and rate law in Eq A, specific rate ($k\ [s^{-1}]$) was estimated. The concentration in the rate law was the initial concentration used by ref [5]. This is because the kinetic data was reported at low conversions (< 20%), and the concentration was therefore close to the initial concentration. The obtained specific rate for 250 °C was used to estimate specific rate for 330 °C, the design temperature for methane oxidation in this study, using Arrhenius equation:

$$k(t) = Ae^{\frac{-E_a}{RT}} (Eq.\ C)$$

The activation energy for each of the considered catalyst was reported in [5]. Using rate constant for 330 °C, assuming no pressure drop in the reactor and isothermal conditions, the volume of catalyst required for the given conversion was estimated using Eq. A. The mass of catalyst was estimated based on its bulk density. Sizing and costing of the reactor was carried out using the same methodology as used for thermal catalytic route.

The costs of the catalysts considered in this analysis is shown in Table S3. The cost of Pd/t is 33.94 M$. The costs of other metals were taken from the references shown in Table S3. The composition of each catalyst was given in Ahmad et al. [5]. The catalyst cost was estimated to be the sum of costs of each element/compound in the catalyst based on their weight fraction. The cost of HAL is 600 $/t [7].

Table S4: Costs of catalysts considered in the sensitivity

Catalyst	Cost ($/t)	Reference
**PdNi@HAL**	309501	https://markets.businessinsider.com/commodities/nickel-price
**PdCo@HAL**	306129.9	https://tradingeconomics.com/commodity/cobalt
**PdCu@HAL**	319662.8	https://markets.businessinsider.com/commodities/copper-price
**Pd@HAL**	312842.5	-

From the results of the sensitivity with different catalysts, we observed Pd@Hal to have a slightly better performance compared to the other catalysts. This contrasts with the performance trend presented at 250 °C in Aliki Marina et al. [8] and Ahmad et al. [5]. We suppose that at steady state and in the absence of other interactions (mass transfer limitations, catalyst deactivation), Pd@Hal is slightly better performing due to its high Arrhenius factor (obtained from kinetic data at 250 °C, please refer to the Table S2, above) despite its high activation energy, low rate constant and reaction rate at 250 °C. It is be noted that this sensitivity doesn't consider mass transfer limitations.

Photocatalytic route

The sensitivity for photocatalysts with different reaction rates was carried out by first estimating the reaction rate constant at each reaction rate.

$$-r''.\rho_b.S \quad = kC_{CH_4} \; (Eq\,.\,D)$$

r'' is the area specific reaction rate in mol/m^2 s. S is the mass specific surface area of the catalyst. ρ_b is the bulk density of catalyst. The following table S4 shows the values/range considered in sensitivity.

Table S5: Values used in sensitivity

Parameter	Unit	Value /range	Reference
r''	$mol/(m^2.s)$	[10^{-9} – 10^{-3}]	[8]
S	m^2/g	[32, 40, 50]	[9], [10]
ρ_b	kg/m^3	833.33	[9]

It is to be noted that as the mass of catalyst decreases with increasing reaction rate, the number of CPC receiver tubes also decreases. This increases superficial velocity significantly in each CPC receiver tube. Superficial velocity can be maintained at reasonable values by increasing cross-sectional area of receiver tubes. This increases the overall surface area in a tube, but that also means more catalyst can be coated in a tube, which in turn reduces the required number of tubes for a given amount of catalyst. To maintain the number of tubes in parallel in such a way so as to have reasonable superficial velocity in each tube, a low thickness catalyst coating is assumed in each tube. Considering that the reactor is sized based on the reaction rate, the specific cost for the cases with and without dehumidifier approaches □740 \$/t-CO_2eq and 165 \$/t-CO_2eq respectively at a reaction rate of 10^{-3} mol/(m^2 s). If the reactor is not sized with the decrease in catalyst mass and keeping the same reactor configuration as the reference case, a more realistic superficial velocity of 0.2 m/s in each tube is achieved. This would however limit the cost decrease to only 1575 \$/t-CO_2eq (for the case with dehumidifier) and □1000 \$/t-CO_2eq (for the case without dehumidifier) at 10^{-3} mol/(m^2 s).

References

1. Sirigina, D.S.S.S., A. Goel, and S.M. Nazir, *Process concepts and analysis for co-removing methane and carbon dioxide from the atmosphere.* Scientific Reports, 2023. **13**(1): p. 17290.
2. Peters, M.S., K.D. Timmerhaus, and R.E. West, *Plant design and economics for chemical engineers*. 5 ed. 2003, New York, NY: McGraw-Hill Professional. 992.
3. Chen, H.-C., F.L. Ng, and Z. Du, *Hybrid laser-TIG welding of dissimilar ferrous steels: 10 mm thick low carbon steel to 304 austenitic stainless steel.* Journal of Manufacturing Processes, 2019. **47**: p. 324-336.
4. Edge, E. *Overall Heat Transfer Coefficient Table Charts and Equation*. Available from: https://www.engineersedge.com/thermodynamics/overall_heat_transfer-table.htm.
5. Ahmad, Y.H., A.T. Mohamed, K.A. Mahmoud, A.S. Aljaber, and S.Y. Al-Qaradawi, *Natural clay-supported palladium catalysts for methane oxidation reaction: effect of alloying.* RSC Advances, 2019. **9**(56): p. 32928-32935.
6. *Halloysite Clay Nanotubes*. Available from: https://phantomplastics.com/functional-fillers/halloysite-filler-gives-unique-properties-to-plastics-and-coatings/.
7. Massaro, M., R. Noto, and S. Riela, *Past, Present and Future Perspectives on Halloysite Clay Minerals.* Molecules, 2020. **25**(20).
8. Aliki Marina, T., S. Joe, T.A. Alexander, F. Shaun, and M.B. Adam, *Exploring the bounds of methane catalysis in the context of atmospheric methane removal.* Environmental Research Letters, 2024. **19**(5): p. 054020.

9. Li, Z., X. Pan, and Z. Yi, *Photocatalytic oxidation of methane over CuO-decorated ZnO nanocatalysts.* Journal of Materials Chemistry A, 2019. **7**(2): p. 469-475.
10. Hoffmann, M.R., S.T. Martin, W. Choi, and D.W. Bahnemann, *Environmental applications of semiconductor photocatalysis.* Chemical reviews, 1995. **95**(1): p. 69-96.
